# **Sustainable production of osteoinductive Co**^**2+**^, **Mg**^**2+**^ **and Mn**^**2+**^ **-substituted apatites particles by one-pot conversion of biogenic calcium carbonate**

**DOI:** 10.1038/s41598-025-94792-7

**Published:** 2025-03-29

**Authors:** Sandra María Cano-Plá, Francesca Oltolina, Francisco Javier Acebedo-Martínez, Raquel Fernández-Penas, Cristóbal Verdugo-Escamilla, Carla Triunfo, Paolo Emanuele Di Simone, Chiara Borsotti, Antonia Follenzi, Gabriele Maoloni, Giuseppe Falini, Jaime Gómez-Morales

**Affiliations:** 1 Laboratory of Crystallographic Studies, Andalusian Earth Science Institute, Spanish National Research Council, Avda. Las Palmeras, No 4, 18100 Armilla, Spain; 2 Dipartimento di Scienze della Salute, Università del Piemonte Orientale, “A. Avogadro” Via Solaroli 17, 28100 Novara, Italy; 3 Department of Chemistry “Giacomo Ciamician”, University of Bologna, Via F. Selmi 2, 40126 Bologna, Italy; 4 Fano Marine Center, The Inter-Institute Center for Research on Marine Biodiversity, Resources and Biotechnologies, Viale Adriatico 1/N, 61032 Fano, Italy; 5 Plant Ascoli Piceno, Finproject S.p.A., 3100 Ascoli Piceno, Italy

**Keywords:** Doped apatites, Magnesium, Manganese, Cobalt, Biogenic calcium carbonate, Osteogenesis, Biological techniques, Materials science

## Abstract

Biogenic CaCO_3_ microparticles obtained from oyster shells *Crassostrea gigas* were used as starting material for synthesizing Co^2+^, Mg^2+^ and Mn^2+^-doped apatite nano-submicroparticles, through a one-step hydrothermal conversion. The conversion was completed at 200 °C for 7 days, yielding metal-doped apatite and whitlockite in percentages of 5.3 wt% when adding Co^2+^, 28.7 wt% for Mg^2+^, and 0 wt% for Mn^2+^. Samples were cytocompatible with murine pancreatic endothelial cells (MS1), murine mesenchymal stem cells (m17.ASC), and murine osteoblast’s progenitors (mOBPs) cells. The analysis by flow cytometry and TEM–EDX revealed strong particle–cell interactions, sustained internalization across m17.ASC and mOBPs cells, and potential progressive apatite dissolution in the cellular environment. Additionally, incubating these cells with the metal-doped samples promoted their osteogenic differentiation without needing an osteogenic differentiation medium. Indeed, the evaluation of gene expression by quantitative real-time PCR, the detection of alkaline phosphatase activity, and the ability to induce the mineralization in the cellular matrix analyzed by alizarin red staining revealed that all particles (and particularly the carbonated apatite and the Mg-doped sample) encouraged the osteogenic commitment. This approach represents a sustainable way to valorize and transform aquaculture and canning industries’ mineral waste (shells) in highly demanded osteoinductive materials.

**Supplementary Information:**

The online version contains supplementary material available at 10.1038/s41598-025-94792-7.

## Introduction

Mollusc shells are hierarchically arranged hybrid organic/mineral composites formed of calcium carbonate (CaCO_3_) crystals embedded within an organic matrix (1–5 wt%), which is composed mainly of proteins and polysaccharides^1^. In 2018, the world production of shelled molluscs, which includes both marine aquaculture and fishery, totalized 17.3 million tonnes^2^. The shells of these molluscs represent a waste by-product^3^ and the vast majority of them are just dumped at sea or thrown in landfills^4^. This practice is not only economically expensive but also poses serious problems for the environment as climate and weather factors cause these shells to degrade and give off bad odors that attract insects and with them spread diseases^5^. Additionally, useful biogenic materials are lost in the process. The recycling of these shells is a clear example of the circular economy because, in addition to being valuable for other sectors, it can provide secondary benefits to fishermen and processors^6^. In this regard, seashells have been used as a source of biogenic CaCO_3_ (bCC) in different fields including calcium supplement for livestock feeding^7,8^, liming agents for soil acidity treatment in agriculture^9^, and as an aggregate substitute in concrete preparation^10,11^.

Besides these applications of low added-value, several studies have shown the feasibility of using bCC from shells of molluscs, i.e. oysters^12^, mussels^13^ or clams^14^, as raw materials in the synthesis of calcium phosphate (CaP) particles of the phase apatite (Ap). The favorable biological properties of this phase, including bioactivity, osteoinductivity, osteoconductivity, non-immunogenicity and non-cytotoxicity, make it suitable for either repairing/regenerating small bone defects or carrier particles for drugs or biomolecules delivery^15^. Moreover, modifying the Ap structure by doping it with different ions allows for new or improved physicochemical properties and biological activity^16^. In this regard, due to the high flexibility of the Ap crystal structure, the anions (OH^−^ and PO_4_^3^) and cation (Ca^2+^) can be substituted with non-metallic and metallic ions^15,17,18^.

It was shown that metallic ions such as Mg^2+^ and Mn^2+^ play an important role in the osseous metabolism. Thus, Mg^2+^ influences all stages of skeletal metabolism being involved in the calcification process, reducing bone fragility, and helping prevent the onset of osteoporosis^19^. The incorporation of Mg^2+^ to the Ap is of great interest in the development of artificial bone substitutes^20,21^. Manganese (Mn^2+^) is involved in the metabolism and development of muscles and bones, potentially aiding in tissue regeneration^22^, although its role in these specific biological processes is still debated^23^. Studies reported that Mn^2+^ can mediate the upregulation of genes involved in osteogenic commitment and improves the formation of the extracellular matrix (ECM) acting as a bioactive chemical species^24^. Doping Ap with Mn^2+^ has proven to be reliable for some biological processes such as biomineralization and bone formation, causing less bone resorption^25^. Additionally, cobalt (Co^2+^), has become appealing for its ability to improve wound healing by reducing bacterial infection and facilitating new blood vessel formation^26^. It was reported that Co^2+^ could simulate hypoxic conditions, which is critical in angio-osteogenesis. This process involves generating cellular oxidative stress, resulting in an upregulation and stabilization of hypoxia inducible factor 1 alpha (HIF-1α), leading to the expression of vascular endothelial growth factor (VEGF), a regulator of angiogenesis in osteoblasts^17,27^. The Co-doped Ap can be considered as a feasible nanoparticles-based device for bone tissue engineering applications.

Several strategies have been developed for the synthesis of Ap using bCC, mostly involving two steps. One approach consists in CaCO_3_ calcination to CaO and CO_2_ by heating the CaCO_3_ up to 900–1100 °C, followed by acid–base titration of hydrated CaO using a phosphate reagent, commonly H_3_PO_4_^13,28–31^. Another method involves wet mechanosynthesis followed by treatment with (NH_4_)_2_HPO_4_ or H_3_PO_4_^32^. The hydrothermal technique was used in a three-step process consisting of calcination of bCC, hydration and carbonation of the resulting CaO to produce precipitated CaCO_3_ particles, and then reaction with (NH_4_)_2_HPO_4_ at 160 °C in an autoclave to produce Ap nanoparticles^33^. A one-step hydrothermal method able to fully transform bCC particles to Ap nano-microparticles was recently developed by our team^34,35^. The method is straightforward, low cost, and environmentally sustainable, i.e. it avoids the calcination step and, therefore, there is neither release of CO_2_ nor use of strong acids or expensive reagents. This study aims to expand the method to the sustainable production of metal-doped Ap particles by converting bCC from oyster shell waste. Essential metallic ions such as Mg^2+^-, Mn^2+^- and Co^2+^ will be used for the proof of concept. To verify the osteogenic performance of the new particles, their biocompatibility and osteoinductive capacity will be tested at all stages of the osteogenic differentiation process using murine osteoblast progenitors, and mesenchymal stem cells.

## Results

### Evolution of the conversion of bCC to metal-doped apatites with the temperature

**Fig. 1:**
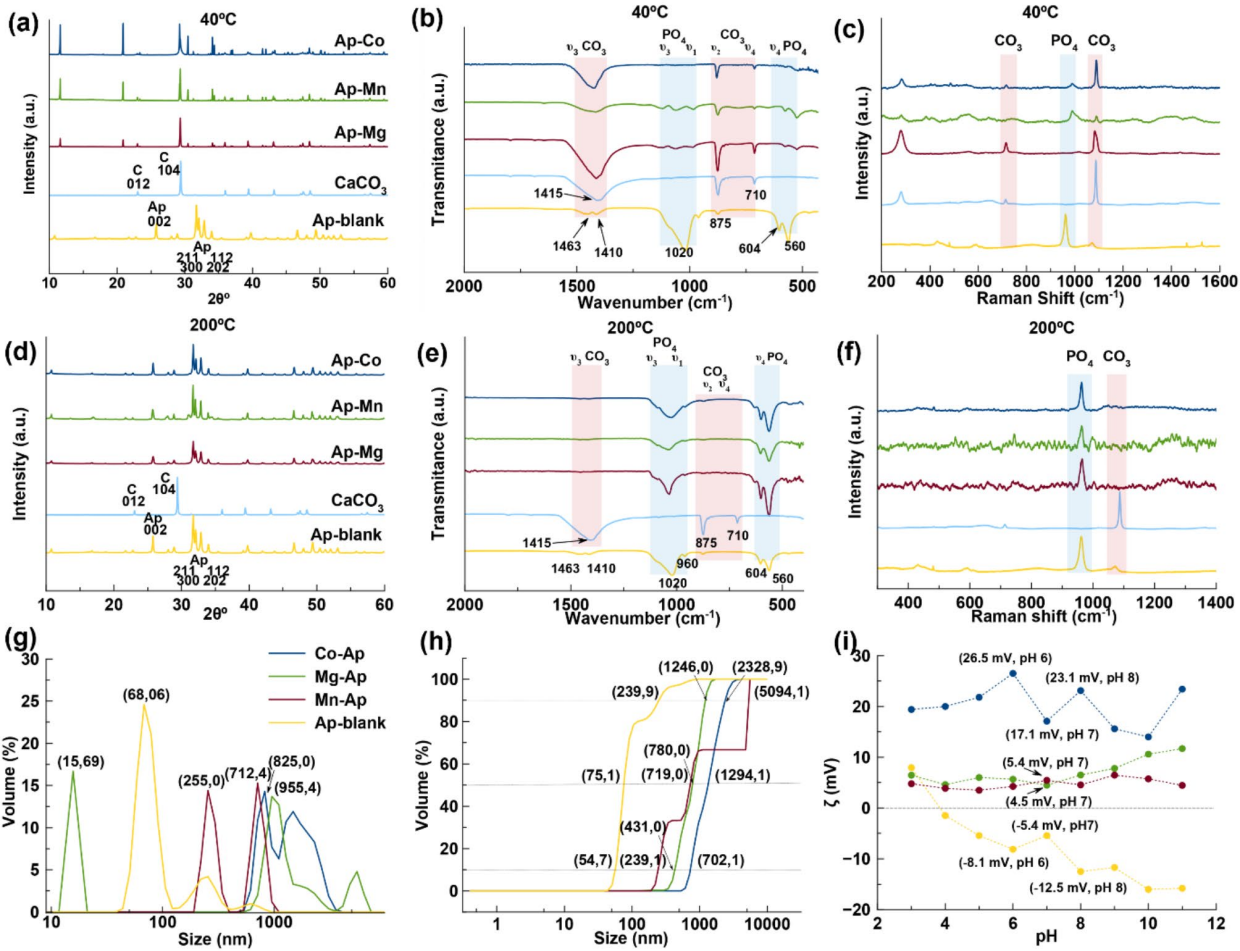
Conversion of bCCP to Mn-, Mg- and Co-doped apatites (Ap-M) at 40 °C and 200 °C analyzed by XRD (**a**,**d**), FTIR (**b**,**e**) and Raman spectroscopy (**c**,**f**), and plots of the corresponding crystal size distributions in volume (**g**) and cumulative volume oversize distribution (**h**) analyzed by DLS, and ζ-potential versus pH (**i**).

The conversion of bCCP with the temperature in the presence of 10 mM Mg^2+^, Mn^2+^ and Co^2+^ was analyzed by X-ray diffraction (XRD), Fourier Transform Infrared spectroscopy (FTIR) and Raman spectroscopy (Fig. 1). The XRD diagrams of samples prepared at 40 °C (Fig. 1a) show the most intense reflections corresponding to CaCO_3_ (calcite phase, PDF 01-086-2339) such as (012) at 2θ = 23.6° and (104) at 2θ = 29.3°, with the presence of CaHPO_4_·2H_2_O (brushite) characterized by the reflection (020) at 2θ = 11.70° (PDF 00-009-0077), and the incipiently formed apatite. This phase is identified by the reflections (002) at 2θ = 25.8° and the quadruplet (211), (112), (300), (202) at 31.77°, 32.19°, 32.90°, and 33.9° respectively (PDF 01-089-5631). At 200 °C (Fig. 1d), the reflections of the CaCO_3_ almost disappeared. Figure S1 (see Supplementary Information) shows the diffractograms from 25 to 200 °C in presence of Co^2+^ (Fig. S1a,b), Mg^2+^ (Fig. S1c,d) and Mn^2+^ (Fig. S1e,f). Regardless of the doping metal, as the temperature increases bCCP progressively converts to CaHPO_4_·2H_2_O (brushite) at 25 and 40 °C, and then into Ap. Moreover, when using Co^2+^ and Mg^2+^ a reflection appeared at ~ 31.3° corresponding to the (02 10) plane of whitlockite (W), likely doped with the metal. This reflection is still visible in the sample prepared with Mg^2+^at 200 °C, and it corresponds to Mg-W, a compound of formula Ca_18_Mg_2_(HPO_4_)_2_(PO_4_)_12_ (PDF 04-009-3397) as reported by other authors^36^. The Rietveld refinement of the XRD diagrams (Table S1) reveals a high amount of Mg-W (~ 30 wt%) in the sample doped with Mg^2+^, while the percentage of W decreases to ~ 5 wt% when using Co^2+^ and to 0 when doping with Mn^2+^.

The chemical composition of these precipitates (Table S1) shows that Co^2+^ is largely incorporated into Ap while Mn^2+^ incorporates a few (~ 1 ppm). When using Mg^2+^ the amount is intermediate (4.3 ppm). Table S2 reports the evolution of phase composition at 120, 160 and 200 °C, revealing the progressive disappearance of bCCP and the decreasing percentage of W, except in the samples doped with Mg^2+^_,_ which show no clear trend.

The characterization by FTIR (Figs. 1b,e, and S2) and Raman (Figs. 1c,f, and S4) confirmed the XRD results. The FTIR spectra of bCC display the band of CO_3_^2−^ at ~ 1415 cm^−1^ (ν_3_) and two minor peaks at 875 cm^−1^ (ν_2_) and 710 cm^−1^ (ν_4_). The Ap (blank) spectrum displays the main vibrational modes of PO_4_^3−^ groups at around 1020 cm^−1^ (ν_3_) and at 560 and 604 cm^−1^ (ν_4_). The presence of the 875 cm^−1^ band in the spectrum of Ap is usual in CO_3_-substituted Ap. At 200 °C (Fig. 1e), the major bands of calcite disappeared, leaving a small remnant at ~ 1419 and ~ 1449 cm^−1^ corresponding to the CO_3_ groups of the Ap, and the above-mentioned band at 875 cm^−1^ (ν_2_CO_3_). A deconvolution of this band reveals three sub-bands (Fig. S3). For Ap-Co, they are located at ~ 880, ~ 872, and ~ 866 cm^−1^; for Ap-Mg at ~ 879, ~ 873, and ~ 868 cm^−1^; and for Ap-Mn at ~ 880, ~ 873, and ~ 868 cm^−1^. These sub-bands are attributed to A-type (CO_3_^2−^ replacing OH^−^), B-type (CO_3_^2−^ replacing PO_4_^3−^) and labile CO_3_^2−^ species located at the surface of the particles^37,38^. The degree of carbonation, determined by the method that compares the intensity of ν_2_CO_3_ band with those of the ν_1_–ν_3_PO_4_ ones^38^, was as follows: 2.2 ± 0.2 wt% for the Ap blank, 2.1 ± 0.2 wt% for Ap-Co, 3.2 ± 0.2 wt% for Ap-Mg, and 1.9 ± 0.1 wt% for Ap-Mn. The FTIR technique does not allow us to reveal if at 200 °C some bCC particles remain unconverted. In contrast, the Raman spectra in the range from 800 to 1200 cm^−1^ (Figs. 1c and S4) show the bands υ_1_(PO_4_) of Ap at ~ 961 cm^−1^^39^, and that of calcite υ_1_(CO_3_) at ~ 1086 cm^−1^^40^, as the most representative signals, indicating that the υ_1_(CO_3_) mode at 200 °C (Fig. 1f) has practically disappeared.

When the metal concentration was increased to 20 mM (Fig. S5), the XRD patterns both at 160 °C and at 200 °C (Fig. S5a,b) show the reflection of W at 31.15° becoming more pronounced and broader, and that of calcite at 29.3° persisting. Only in the Ap-Mn sample, the W reflection has disappeared. This was also observed in the Raman spectra at 200 °C (Fig. S5f), where the signal at 1086 cm^−1^ is visible. The finding could be due to the slowdown of the conversion of bCC to CaP, caused by passivation of the bCC particles surface by excessive metal accumulation. Figures 1g,h display the crystal size distributions (CSD) plotted in both volume and cumulative volume. These latter reveal the percentiles D_10_, D_50_, and D_90_ (horizontal lines)^34^. For Ap blank, there is a peak at 68 nm that stands out in volume compared to the others. For the doped samples, however, the first peak is at 15.7 nm when the metal is Mg, 255 nm for Mn, and 825 nm for Co (Fig. 1g). When considering the cumulative volume, only Ap blank presents the D_10_ and D_50_ (median size) in the nanometer range, while those for M-doped CaP fall in the submicrometer and micrometer range, due to the presence of larger particles and larger aggregates. The D_90_ is fully influenced by aggregation in all samples (Fig. 1h).

Figure 1i illustrates the evolution of ζ-potential with pH in aqueous suspensions. This was examined to determine the tendency of the particles either to disperse or aggregate. For Ap blank, we can see that the ζ-potential decreases to ~ − 5.4 (pH 7) and − 15 mV (pH 11). This indicates that the repulsive forces between particles are stronger than the attractive ones, due to the increase in negative surface charges (OH^−^), thus causing the colloid to disperse. This behavior was even more pronounced for the bCCP, reaching ζ-potential values of − 15.1 (pH 7) and − 31.2 mV (pH 11) (Fig. S6). In contrast, metal-doped CaP samples display positive ζ-potentials, likely due to the excess of cations onto the surface of the particles. This caused a charge reversion, which was greater in the case of Co^2+^. Indeed, Ap-Co showed the highest ζ-potentials, reaching values of ~ +25.4 mV (pH 6) and ~ + 17.1 mV (pH 7), in agreement with the highest metal concentration (7.7 ppm). The ζ-potentials of the Mg- and Mn-doped samples are very low at physiological pHs (+5.4 and +4.5 mV at pH 7), indicating that particles are practically uncharged. Under these conditions, electrostatic repulsions are less relevant and the colloidal suspensions are less stable. The dispersion of the colloidal particles is essential when they are used as carriers of chemotherapeutic or anti-inflammatory drugs^41,42^ but a priori this condition seems not be necessary for their use as an osteoinductive implantable material.

### Morphological characteristics of the metal-doped CaP samples

**Fig. 2:**
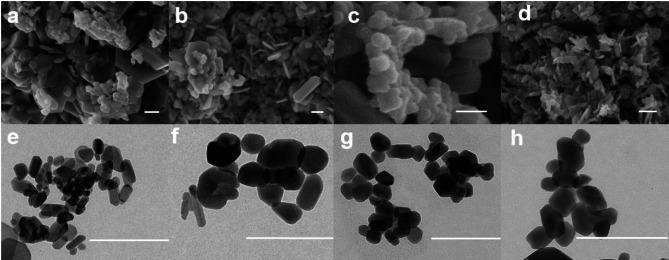
FESEM (**a**–**d**) and TEM images (**e**–**h**) of Ap-blank, Ap-Co, Ap-Mg and Ap-Mn particles prepared at 200 °C. Scale bars = 200 nm.

The microscopic examination of the samples by field emission scanning electron microscopy (FESEM) (Fig. 2a–d) shows the presence of anisometric submicronic (L < 1000 nm) particles of different shapes (hexagonal platy-shaped, prisms, cubes, rods) along with faceted nanoparticles (L < 100 nm), the latest also inspected by TEM (Fig. 2e–h). For the sake of simplicity, here we report the longest average dimension (L) of both the larger particles and the nanoparticles, along with the corresponding standard deviations. Thus, for the Ap-blank (Fig. 2a,e), L were 460 ± 102 nm and 48 ± 20 nm; for Ap-Co (Fig. 2b,f), L = 217 ± 38 nm and 73 ± 18 nm; for the Ap-Mg sample (Fig. 2c,g), L = 360 ± 32 nm and 57 ± 6 nm; and for the Ap-Mn (Fig. 2d,h), L = 180 ± 19 nm and 47 ± 8 nm. The precursor bCC particles have the longest dimensions (L up to 45 µm) (Fig. S7).

In the series of FESEM images of Fig. 2, only the sample labeled “Ap-Mg-” (Fig. 2c) composed of Ap-Mg (~ 70 wt %) and Mg-W (29 wt %) clearly displays two types of major morphologies, the faceted nanorods and hexagonal prisms.

**Fig. 3:**
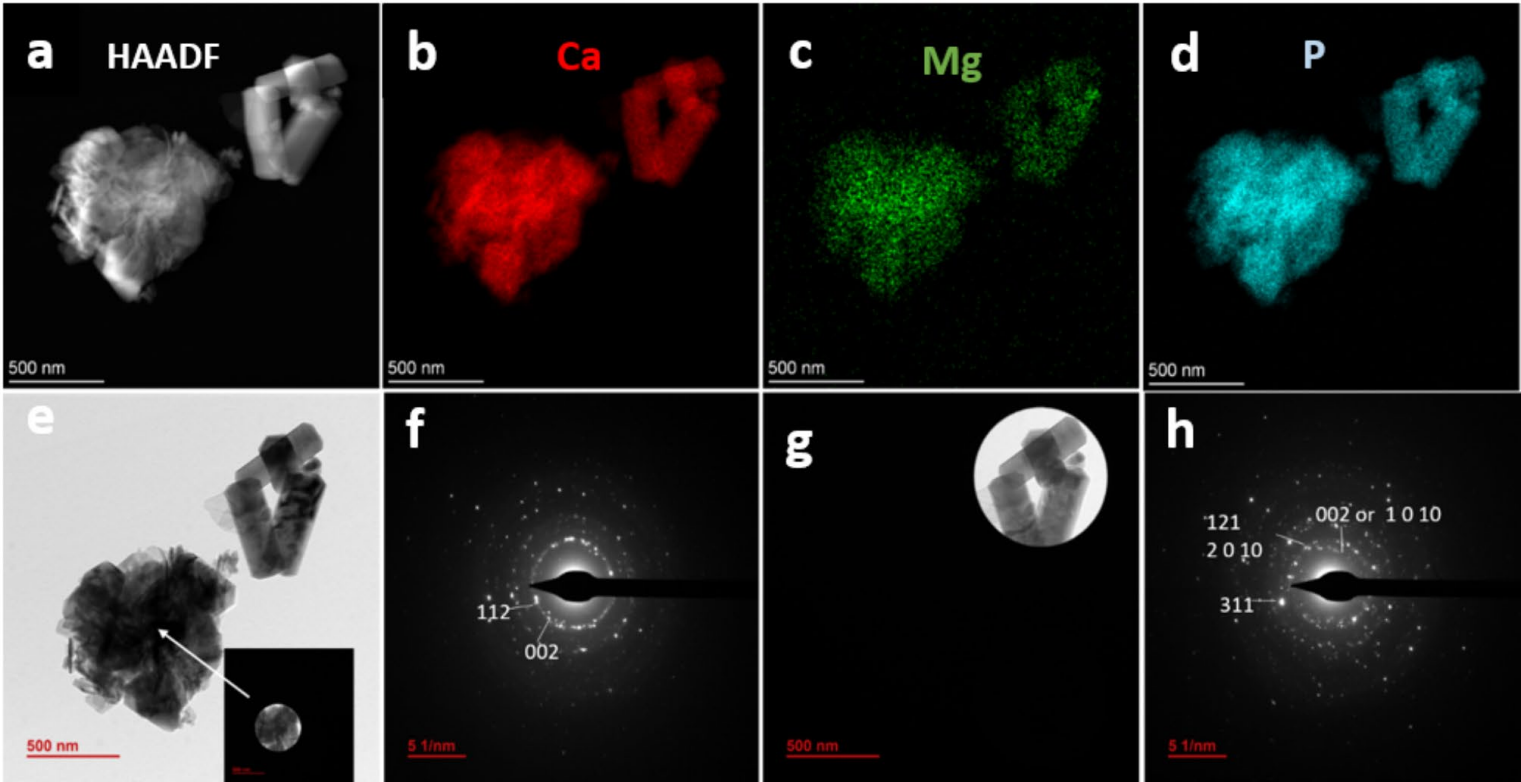
(**a**) HAADF-STEM micrograph of the sample labeled Ap-Mg showing nanorods and hexagonal prisms. (**b**–**d**) EDX element mappings of Ca, Mg, and P. (**e**–**h**) TEM images and SAED patterns of nanorods and prisms.

This sample was further analyzed by HRTEM. The high-angle annular dark field-scanning transmission electron micrograph (HAADF-STEM) image (Fig. 3a) and elemental EDX mappings of Ca, Mg and P (Fig. 3b–d) show that both morphologies are doped with Mg^2+^ ions. The selected area electron diffraction pattern (SAED) of the nanoparticles (Fig. 3e,f) shows the indexed reflections of planes (002) and (211) of the Ap. In the same sample, the SAED pattern of the prisms (Fig. 3g,h) depicts some rings and spots associated to the d-spacings 2.87 Å, 2.74 Å, 2.69 Å, 2.66 Å, 1.94 Å, 1.91 Å, and 1.67 Å, which correspond to the planes (0 2 10), (1 2 8), (0 3 6), (1 1 12), (1 4 3), (1 1 18) and (5 0 8) of Mg-W (PDF 04-009-3397). Some of the rings may be also associated with planes (002), (121), and (311) of the Ap, which indicates the presence of both phases in the selected area.

### Biological properties

#### Cells and particles–cells interactions

To evaluate the cytotoxicity of the metal-doped samples, three murine cell types (MS1, m17.ASC and mOBPs) were used, and a MTT assay was performed after 3 days of incubation. Figure S8 shows the cell viability outcomes for particles concentrations ranging from 0.1 to 100 µg/mL, and in the presence of soluble doxorubicin (Doxo), a chemotherapy drug used as an internal control. When compared with the untreated cells (CTRL−), doped-particles exhibited excellent cytocompatibility in all cells since the viability was higher than 80%. Only when MS1 cells were treated with the highest dose of the Ap-Co sample, a reduced viability was observed (about 75%) but still acceptable, being higher than 70% which is the allowed limit by ISO 10993-5:2009^43^. As expected, cell viability in the presence of Doxo decreases below 50% in all the tested concentrations.

Moreover, to evaluate the cellular uptake of the metal-doped CaP particles in m17.ASC and mOBPs cells, we assessed particles interaction with cells through transmission electron microscopy (TEM) and flow cytometry, detecting changes in cellular morphology and complexity.

**Fig. 4:**
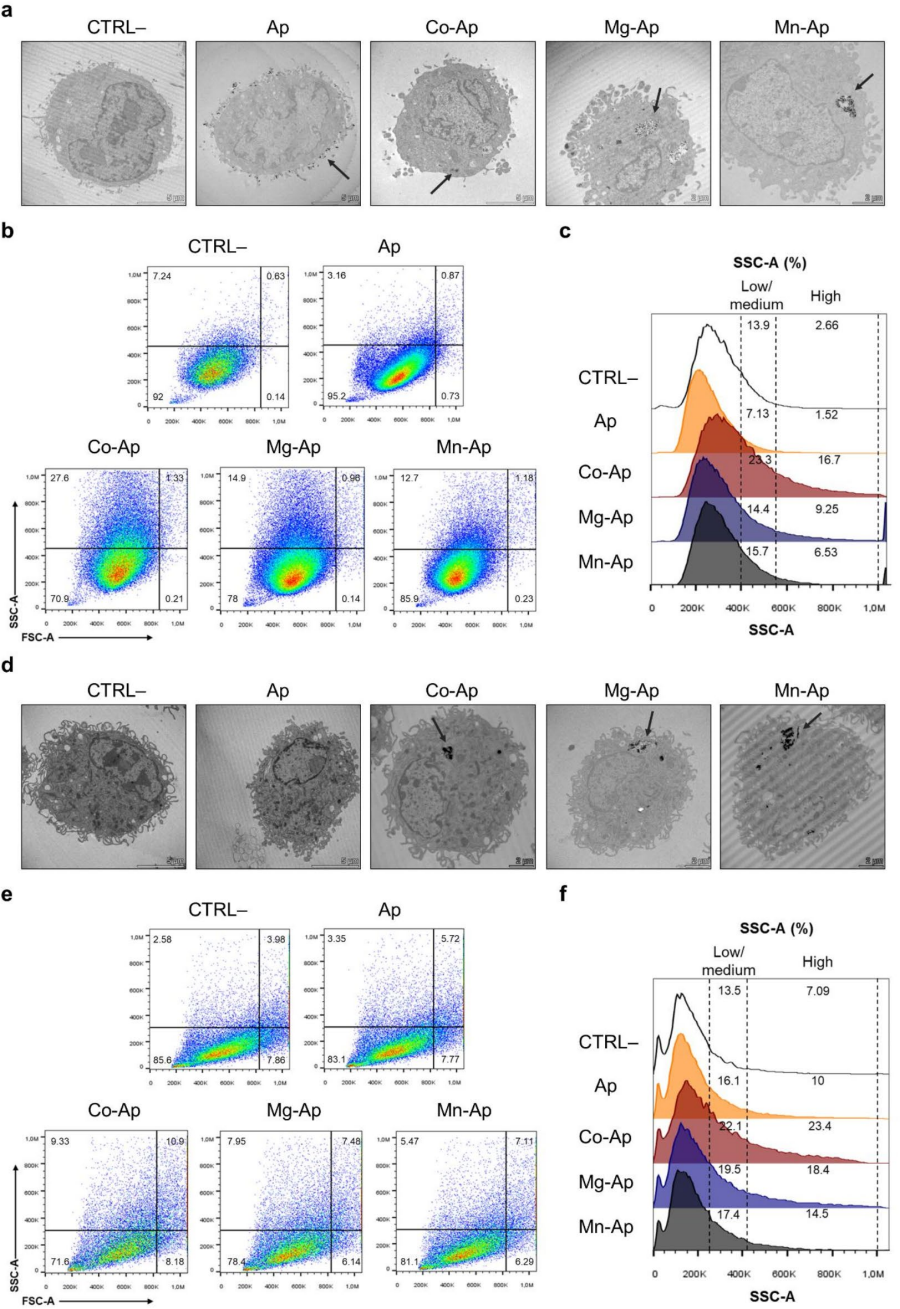
Interaction of 25 µg/mL particles with m17.ASC and mOBPs cells analyzed by TEM and flow cytometry after 24 h of exposure. Representative TEM photomicrographs show particles internalization in m17.ASC (**a**) and mOBPs (**d**) cells compared to untreated controls (CTRL−). Photomicrographs are representative of alternate serial cuts of the cell pellets of each sample. Scale bar: 2–5 µm. Representative dot plots show changes in morphology, based on physical parameters such as size (FSC-A) and complexity (SSC-A), in m17.ASC (**b**) and mOBPs (**e**) cells treated with particles, compared to untreated controls (CTRL−). Representative histograms display the percentage of cells with increased complexity, categorized by low/medium or high SSC-A for m17.ASC (**c**) and mOBPs (**f**) cells relative to the untreated control (CTRL−).

As reported in Fig. 4a and d doped particles were clearly detectable in both cell types after 24 h of exposure. Metal-doped CaP particles were internalized by m17.ASC and mOBPs cells mainly localized in the cytoplasm, while Ap particles displayed a delayed interaction pattern since they are detected on the cell membrane. In accordance, cells treated with particles displayed distinct morphological changes compared to untreated controls, which predominantly showed low SSC-A profiles (92%), indicating cellular complexity. In m17.ASC cells, exposure to particles significantly increased complexity, with Ap-Co inducing the most pronounced shift (70.9% low SSC-A), followed by Ap-Mg (78%) and Ap-Mn samples (85.9%), while Ap did not induce noticeable changes. (Fig. 4b). The distribution of cells exhibiting low, medium, and high SSC-A mirrored these observations (Fig. 4c). The same trend was displayed by mOBPs cells when treated with the particles (Fig. 4e,f). Interestingly, by day 7 both cell types showed more pronounced changes in their morphological profiles: m17.ASC cells particles-treated showed a dramatic shift in complexity, with the low SSC-A population decreasing to 60–70% across all treatment groups compared to 96.4% in controls (Fig. S9a). Notably, the high SSC-A population increased substantially, reaching 76.5–80.7% in treated cells compared to just 3.56% in controls (Fig. S9b), indicating significant cellular internalization of particles over time. Similarly, mOBPs at day 7 revealed sustained alterations in cellular complexity, with all treatment groups maintaining approximately 64–68% low SSC-A populations compared to 83.2% in controls (Fig. S9c). The high SSC-A population increased to 26.5–28.5% in treated cells versus 13.8% in controls (Fig. S9d), suggesting persistent particles interaction with the cells.

**Fig. 5:**
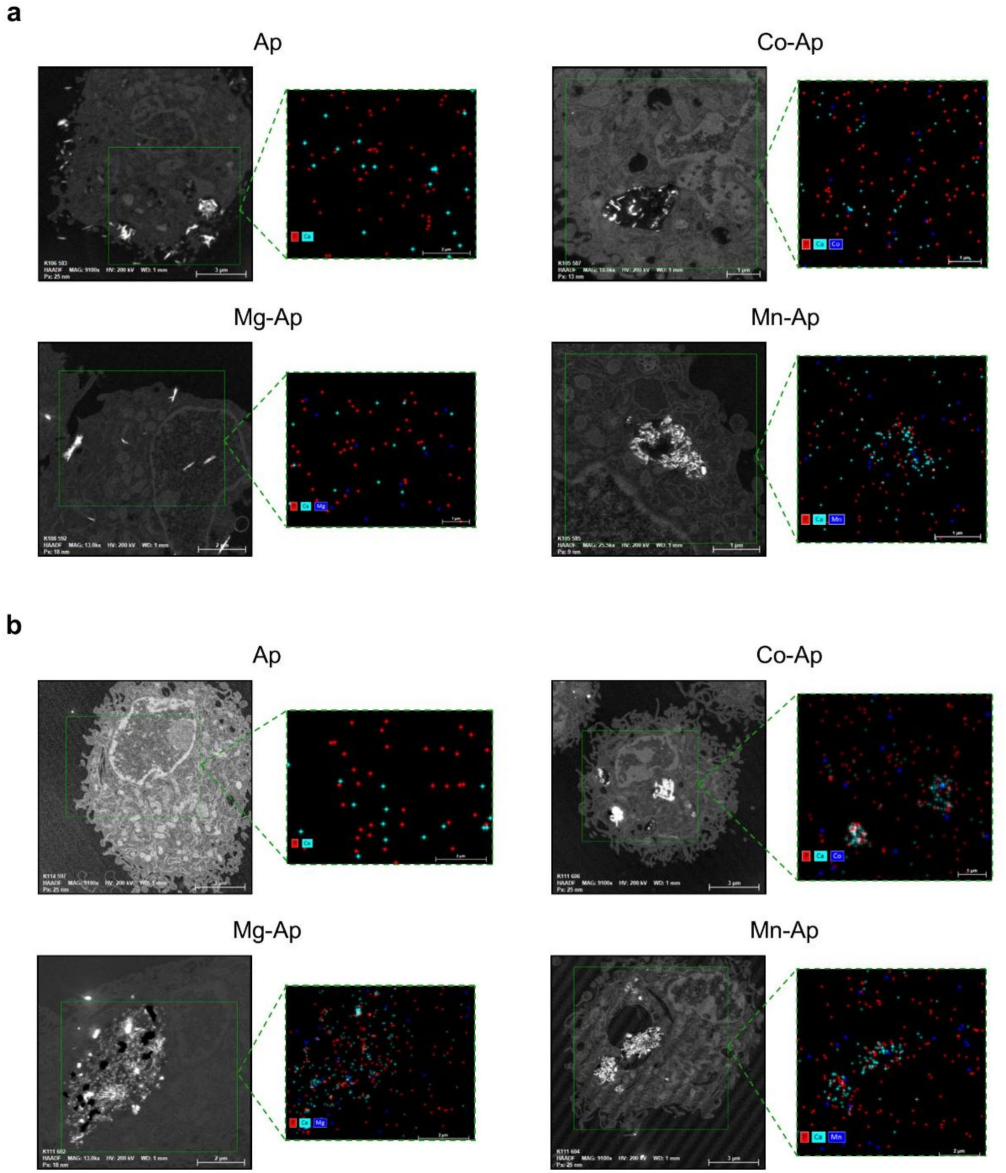
EDX element mappings of M-doped CaP particles treated m17.ASC (**a**) and mOBPs (**b**) cells after 24 h exposure with the particles. Points correspond to characteristic EDX emissions from detected elements. Scale bar: 2–5 µm.

Moreover, energy dispersive X-ray spectroscopy (EDX) analysis coupled to TEM was performed to evaluate the intracellular element composition of m17.ASC and mOBPs cells following 24 h exposure with particles. The analysis revealed distinct element profiles that correspond to the specific composition of each tested particle formulation. Interestingly, cells treated with Ap demonstrated characteristic calcium and phosphate signals, confirming the presence of internalized particles in accordance with TEM results (Fig. 5a,b). Notably, both cell types exposed to metal-doped CaP particles formulations showed specific intracellular signals corresponding to their respective dopant elements corroborating cellular internalization. Furthermore, the relative intensities of Ca, P and M signals across treatment groups suggest progressive dissolution of the metal-doped CaP particles within the cytoplasm.

#### Osteogenic differentiation assays

To evaluate the osteogenic behavior of the here-mentioned metal-doped CaP samples, gene expression analysis, alkaline phosphatase and Alizarin Red staining were performed, demonstrating the osteogenic differentiation potential of murine mesenchymal stem cells (m17.ASC) and murine osteoblast progenitor (mOBPs) cells.

##### Gene expression

To assess the ability of m17.ASC and mOBPs cells to differentiate through the osteogenic phenotype, cells were treated with 25 µg/mL of particles for 10 days. The preliminary osteogenic commitment was analyzed by quantitative real-time PCR (q-RT-PCR) assessment of the bone morphogenetic protein 2 (BMP2), type 1 collagen A (COL1A1–COL1A2), bone gamma-carboxyglutamate protein (BGLAP—osteocalcin), secreted phosphoprotein 1 (SPP1—osteopontin) and receptor activator of nuclear factor kappa B ligand (RANKL) genes (see Table S3 of SI).The osteogenic medium served as a positive control^44^.

**Fig. 6:**
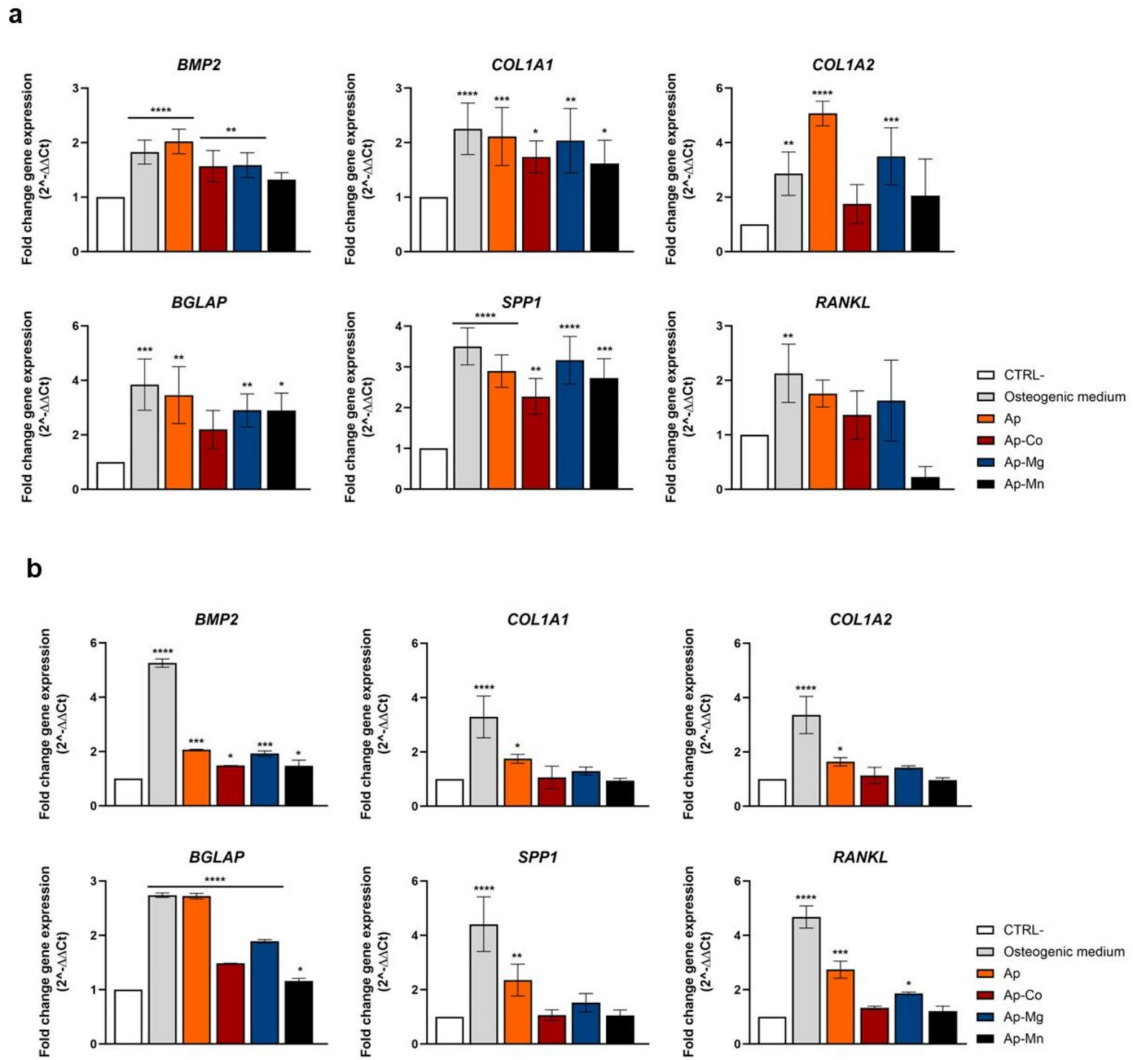
Relative-fold expression of marker genes in m17.ASC (**a**) and mOBPs (**b**). Histograms showing mRNA levels of bone morphogenetic protein 2 (BMP2), type 1 collagen A (COL1A1 and COL1A2), bone gamma-carboxyglutamate protein (BGLAP—osteocalcin), secreted phosphoprotein 1 (SPP1—osteopontin) and receptor activator of nuclear factor kappa B ligand (RANKL) genes detected by q-RT-PCR following the treatment of Ap nanoparticles. Gene expression was expressed as fold change relative to untreated cells (CTRL−). Data are expressed as the mean ± SD of three independent experiments using one-way ANOVA with the Dunnet’s comparison post-test versus CTRL−. (*p < 0.05; **p < 0.01; ***p < 0.001; ****p < 0.0001).

The results (Fig. 6) showed that metal-doped samples induced BMP2 expression in m17.ASC (Fig. 6a) and in mOBPs cells (Fig. 6b). COL1A1 and COL1A2, early markers of pre-osteoblasts differentiation, were up-regulated in m17.ASC cells after particle treatment, while in mOBPs cells only the osteogenic medium and Ap increased mRNA levels^45^. Similarly, the BGLAP gene, which is mainly expressed by osteoblasts at early stages of differentiation, was up-regulated in both cell types when treated with the osteogenic medium and with the native Ap as well as the metal-doped samples. The late-stage marker SPP1 expression^46^ was modulated more than twice compared with the control group in both cells when treated with the osteogenic medium and with the native Ap. The same pattern was detected in m17.ASC cells treated with metal-doped CaP particles. Only the osteogenic medium was able to induce the expression of RANKL in m17.ASC, while in mOBPs, not only this treatment upregulated this gene, but also when cells were treated with Ap and Mg-doped samples.

##### Alkaline phosphatase (ALP) staining

**Fig. 7:**
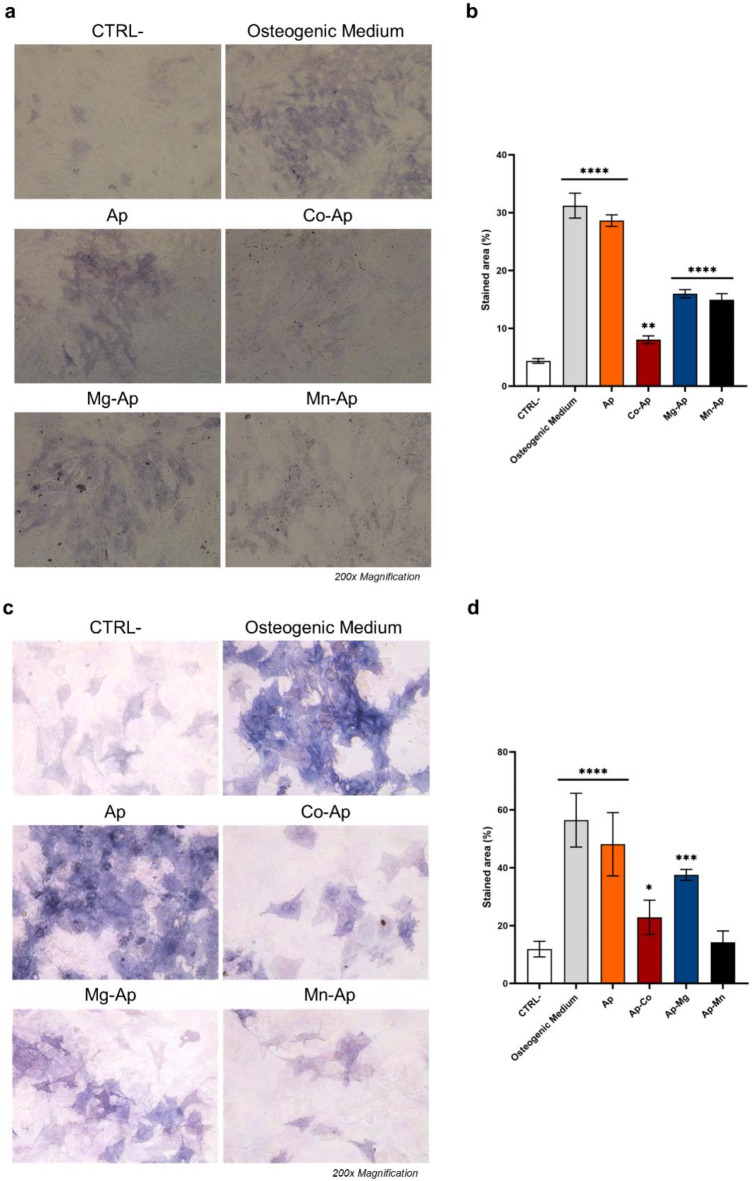
Effect of the particles on the activity of ALP enzyme on m17.ASC and on mOBPs cells. Optical microscope images of the stained m17.ASC (**a**) and mOBPs cells (**c**), magnification 200×. Quantitative analysis of ALP activity on m17.ASC (**b**) and mOBPs cells (**d**) obtained by counting stained area with ImageJ software, statistic was calculated using one-way ANOVA with Dunnett’s comparison post-test versus CTRL− (*p < 0.05; **p < 0.01; ***p < 0.001; ****p < 0.0001).

Alkaline phosphatase (ALP) staining is a technique widely used to evaluate the activity of the ALP enzyme, which serves as an early indicator of osteogenic differentiation. Figure 7a and c show m17.ASC and mOBPs cells, respectively, after being treated with the particles for 14 days and then stained for ALP.

Quantitative analyses (Fig. 7b,d) demonstrated that Ap induces a similar osteogenic effect as the osteogenic medium. When compared with the untreated cells, the Ap-Mg and Ap-Mn samples also presented significantly higher levels of ALP activity, indicating a discrete rate of osteogenic differentiation. However, these results suggest that, under the tested experimental conditions, Ap osteogenic ability is reduced by the introduction of metal ions in its crystalline structure and probably by the presence of the W-M phase.

##### Alizarin red staining (ARS)

**Fig. 8:**
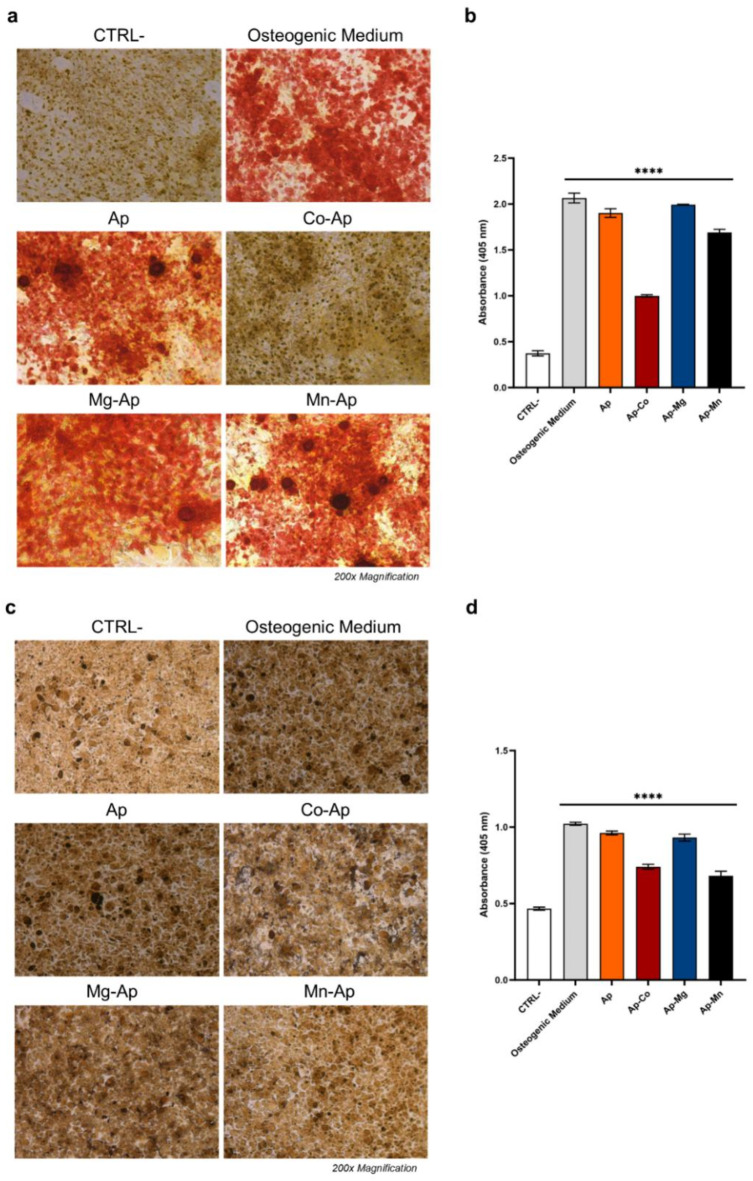
Effect of the particles on calcium phosphate deposits in the cell matrix of m.17.ASC and mOBPs cells stained with ARS and quantified. Optical microscope images of the stained m17.ASC (**a**) and mOBPs cells (**c**), magnification 200×. Quantitative analysis of ARS obtained by dissolving calcium phosphates deposits of m17.ASC (**b**) and mOBPs cells (**d**), and the absorbance was then measured. Statistics were calculated using one-way ANOVA with Dunnett’s comparison post-test vs. CTRL− (****p < 0.0001).

Besides ALP, Alizarin red staining (ARS) is a major indicator of the osteogenic differentiation process since it allows observing and quantifying CaP deposits in the cell matrix. Figure 8a and b shows the optical microscopy images of the ARS experiments after 14 days of incubation on both m17.ASC and mOBPs. The quantitative analysis of the experiment for the two cells in Fig. 8c and d clearly shows that mineralization did not occur in non-treated cells (CTRL−), while cells treated with the osteogenic differentiation medium present the highest rate of mineralization. Concerning the treatment with the particles, undoped Ap presented the highest rate of mineralization, almost at the same level as the osteogenic medium. The metal-doped samples were also able to induce a significant increase in the mineralization matrix without adding extra osteogenesis stimulators.

## Discussion

Metals are essential trace elements for life, but their concentration can impact the mineral and enzymatic status in organisms posing a potential threat to human health^47^. In bone, Ap is the main mineral constituent and Mn^2+^, Co^2+^ and Mg^2+^ have been reported to exert some essential functions in bone metabolism^17,22,47,48^. This study explores the conversion of bCC into Ap and metal-doped Ap (Ap-Co, Ap-Mn and Ap-Mg) particles using the one-pot hydrothermal method^34^. The aim is twofold: to recycle a waste material largely disposed of in landfills by a sustainable method, and fabricating new ceramic particles with osteoinductive ability. This approach aligns with the principles of the circular economy and is novel, as there are no reports, to our knowledge, on the production of these metal-doped CaP samples from bCC.

The study shows a gradual transformation of bCC to brushite and then to metal-doped Ap as the temperature increases. Almost complete conversion to metal-doped Ap micro-nanoparticles occurs at 200 °C. Additionally, the presence of W is observed as a secondary phase when using Co^2+^ and Mg^2+^. These results differ from the preparation of Ap in the absence of metals by this method where complete transformation occurred at 160 °C without any additional phase. Moreover, the metal-doped Aps present CO_3_^2−^ doping the structure in A- and B-position, and labile CO_3_^2−^ ions, a characteristic of biomimetic Ap^18,49^. Their higher propensity to aggregate compared to the Ap particles is essentially due to the heterogeneity of particle size, and to the low ζ-potential values of their aqueous suspensions. In this regard, the positive ζ-potential due to surface charge reversion is explained because besides doping the bulk crystal structure, part of the metals are accumulated at the outermost layers of the particles surface, particularly in the case of Ap-Co, which is the most heavily doped sample.

An interesting finding by inspecting the metal-doped Ap nanoparticles by TEM is that all nanoparticles, regardless the metal tend to acquire a flatter, plate-shaped morphology, instead of the elongated morphology more characteristic of the Ap blank. This is more evident for Ap-Mg nanoparticles (Fig. 2g) and indicates the growth along the *a*- and *b*-directions is favored rather than along the *c*-axis due to the incorporation of the metals. The morphological scenario of samples containing Mg^2+^ and Mn^2+^ ions is enriched by the appearance of W, being not viable to univocally assign a morphology to one of both phases, even when comparing with morphologies of the same compound reported in the literature^50^.

The cytocompatibility of the derived apatite samples was assessed through MTT assay on three cell types, MS1 (murine endothelial pancreatic cells), m17.ASC (murine mesenchymal stem cells) and mOBPs (murine osteoblast progenitor cells), after a 3-day incubation with concentrations ranging from 0.1 to 100 µg/mL. The results were remarkably positive, with no significant toxic effects found across all tested particles concentrations when incubated with MS1 and m17.ASC. Indeed, the cell viability in these cells consistently exceeded 85% under these conditions. Conversely, mOBPs had a more sensitive response than MS1 and m17.ASC, especially when exposed to the highest particle’s concentration of 100 µg/mL. This can induce oxidative stress, leading to overproduction of reactive oxygen species and damage to cellular components like proteins, lipids, and DNA. Cobalt-based nanoparticles can also contribute to inflammation in human mononuclear cells and neutrophils by releasing pro-inflammatory cytokines^51^.

Indeed, this reduced viability can be caused by direct cellular uptake of doped-particles or dissolution of the Ap-Co particles, leading to an elevated concentration of Co^2+^ in the medium^52^. Hence, Co^2+^ can induce oxidative stress in eukaryotic cells^53^ leading to overproduction of reactive oxygen species (ROS) and damage of cellular components such as proteins, lipids, and DNA. Also, cobalt-based nanoparticles have been found to contribute to inflammation process in human mononuclear cells and neutrophils by releasing pro-inflammatory cytokines^51^.

Although manganese plays a crucial role as a cofactor of enzymes required for specific biological functions such as energy metabolism, blood clotting, and bone development, increasing its dosage triggers cellular toxicity^54^. This event is attributed to oxidative stress leading to lipid peroxidation at the mitochondrial level. Excessive Mn^2+^ ions compete with iron for metal transporters, releasing reactive Fe^2+^/Fe^3+^ ions, which produce hydroxyl radicals causing enzyme degradation, DNA fragmentation, and lipid peroxidation. Thus, Mn-induced oxidative stress parallels Fe-mediated ROS generation, ultimately resulting in cell death. Nevertheless, it is noteworthy that under all experimental conditions, cell viability consistently remained above 70%. This finding is significant, as it exceeds the 70% threshold established by the ISO 10993-5:2009 guidelines. This interesting result confirms the broader acceptability of the doped-calcium-based particles for cytocompatibility studies in biomedical and biotechnological applications.

Moreover, by flow cytometry we demonstrated that particles were internalized by m17.ASC and mOBPs cells after 24 h exposure, showing variations in cell shapes, sizes and increased granularity. Interestingly, these patterns increased overtime, highlighting persistent interaction within the cells by sustained shifts in physical parameters. TEM analysis provided a direct visualization of these interactions also corroborated by EDX analysis. In addition, EDX element mappings also highlighted a progressive dissolution of the apatite matrix within the cytoplasm. The consistency of these results across both cell types strengthens our observations regarding particles-cell interactions and their temporal dynamics, suggesting that their elements might play a key role in osteogenesis^55^.

In another set of experiments the ability of the particles to induce osteogenic differentiation in both cell types, was evaluated by gene expression analysis, detection of alkaline phosphatase (ALP) activity and the ability to induce mineralization in the cellular matrix.

Intriguingly, it was observed that m17.ASC and mOBPs cells exhibited a sequential upregulation of key marker genes corresponding to various stages of osteogenic differentiation and maturation when cultured with metal-doped Ap nanoparticles. Notably, this occurred without the addition of any osteogenic factors showing comparable efficacy to the treatment with osteogenic media.

We evaluated bone morphogenetic protein 2 (BMP2), a key component in bone development and a member of the TGF-β superfamily. BMP2 is crucial for bone formation, fracture healing, and spinal fusion by binding to cell-surface receptors and inducing osteoblast differentiation^56^. BMP2 was upregulated in m17.ASC and mOBPs, driving the expression of the major matrix molecules in bones, type-I collagen (COL1A1 and COL1A2), early indicators of osteoprogenitor cells^57^. COL1A1, COL1A2, with ALP are essential in osteogenesis by acting as structural components forming type I collagen, which provides the tensile strength necessary for bone matrix formation. Also, COL1A1 and COL1A2 play an integral role in the mineralization phase of bone development^58^. Interestingly, all particles significantly upregulated COL1A1 expression in m17.ASC cells compared to the ones in culture indicating their ability in the modulation of this gene. However, COL1A2 was only upregulated in cells treated with Ap and Mg-doped particles. The mOBPs cells expressed high levels of COL1A1 and COL1A2 only when treated with osteogenic medium and Ap nanoparticles, likely because type I collagen genes are early osteogenesis markers and their expression decreases over time, especially in already committed cells like mOBPs compared to mesenchymal stem cells from adipose tissue^46^. Analysis of the bone gamma-carboxyglutamate protein (BGLAP) gene, also known as osteocalcin, revealed a similar pattern. BGLAP is considered a middle-to-late-stage marker of osteogenic differentiation^55^ and particles significantly increased BGLAP expression in m17.ASC and mOBPs cells. Similarly, secreted phosphoprotein 1 (SPP1) gene expression is upregulated by all the particles in m17.ASCs, while only osteogenic media with the *naïve* Ap NPs affect its expression in mOBPs cells. This particles-mediated SPP1 upregulation is notable, as SPP1 encodes osteopontin (OPN), a late-stage marker that promotes bone formation and mineralization. Hence, this glycoprotein is related to osteogenesis and anchoring of osteoclasts to the bone-remodeling matrix via binding to the vitronectin receptor. Moreover, SPP1 is present not only in osteoblasts and the mineralized bone matrix, but also in intramembranous ossification, and it enhances osteoblastic differentiation and proliferation^59^. Furthermore, we analyzed the receptor activator of nuclear factor kappa B ligand (RANKL) gene, known for its role in osteoclast activation through its induced expression by osteoblastic cells^60^. The multifaceted regulation of RANKL expression is complex, indeed, several factors, including vitamin D3 and glucocorticoids act as influencers of RANKL expression making it a central player in the intricate interplay between bone formation and resorption.

Additionally, Wu et al. discussed the potential role of nanomaterials, including Ap nanoparticles in modulating osteoblast-osteoclast interactions highlighting this intricate crosstalk mediated by various signalling molecules, including RANKL^61^. Only osteogenic medium induced RANKL gene expression in m17.ASC compared to mOBPs in which the expression was also modulated by Ap and Ap-Mg samples indicating a more prone attitude of these cells to undergo to osteogenic differentiation. In additional assays, we proved that m17.ASC and mOBPs cells undergo osteogenesis and mineralization in response to metal-doped Ap particles. Several studies have shown that calcium-based nanomaterials boost ALP enzyme activity, essential to the osteogenesis process, peaking within 2 weeks^62^. During a 14-day incubation, both particle-treated and untreated m17.ASC and mOBPs cells exhibited a significant ALP positive staining, with *naïve* Ap NPs showing the best cellular response^34^. Among metal-doped particles, Mg-doped samples performed best.

Moreover, the presence of calcium salt deposits in the bone matrix—detected by ARS—is regarded as a distinguishing feature of osteogenesis that occurs during the last phase of osteoblast differentiation. In fact, we observed that in both cell lines treated with metal-doped particles, the mineralized matrix was well detectable as a red nodule-like staining.

Finally, this study suggests that even without osteogenesis stimulators, *naïve* and metal-doped CaP particles, especially Mg-doped ones, enhance osteogenic capacity in osteoblast-committed cells.

## Conclusions

Complete conversion of bCCP to metal-doped CaP phases (Co-, Mg- and Mn-doped Ap nano-microparticles containing CO_3_^2−^ ions within its crystal structure, accompanied by whitlockite as minor phase, i.e. 5.3 wt% when using Co^2+^, 28.7 wt% for Mg^2+^, and 0 wt% for Mn^2+^) has been successfully achieved using a one-step hydrothermal method at temperatures around 200 °C. The doped samples present plate-shaped and rod-like morphologies with average particle size larger than that of undoped Ap samples, and a higher aggregation trend. All samples show excellent cytocompatibility up to 3 days when incubated with murine pancreatic endothelial cells (MS1), mesenchymal stem cells (m17.ASC) and murine osteoblast´s progenitors (mOBPs) cells. Furthermore, our comprehensive TEM–EDX and flow cytometry analysis demonstrated robust particles-cell interactions and sustained internalization in m17.ASC and mOBPs cells, together with a progressive apatite matrix dissolution in the cytoplasm. Our findings indicate that metal-doped-CaP particles, especially those doped with Mg^2+^, promote osteogenesis in m17.ASC and mOBPs. mRNA levels of osteogenic markers such as BMP2, COL1A1, COL1A2, BGLAP, and SPP1 are upregulated, suggesting that these particles can stimulate different phases of osteogenesis and maturation without the requirement for extra osteogenic differentiation medium. The potential of the metal-doped CaP particles to facilitate bone regeneration is further shown by the increase in ALP activity and the ability to induce the mineralization in the cellular matrix analyzed by ARS. Finally, we plan to further develop and test these particles combined with nucleic acids or viral vectors expressing cytokines or other regulatory molecules promoting both cell survival or able to limit inflammation for regenerative medicine approaches.

## Methods

### Reagents and preparation of biogenic CaCO_3_ powder (bCC)

Oyster shells from the species *Crassostrea gigas* were provided by F.lli Terzi (Palosco, BG, Italy). The shells were washed with tap water, then immersed in 5% v/v NaClO solution for 24 h to remove the surface organic residues, washed again first with tap water and then with deionized water and finally air-dried. Next, the shells were crushed by a hammer mill and the resulting biogenic CaCO_3_ powder (hereafter bCCP) sieved at Ø < 45 µm. Potassium phosphate monobasic (KH_2_PO_4_, MW = 136.08 g/mol, ACS reagent, 99% purity), magnesium chloride hexahydrate (MgCl_2_·6H_2_O, MW = 203.30 g/mol, 99% purity), manganese chloride hexahydrate (MnCl_2_·6H_2_O, MW = 197.91 g/mol) and cobalt chloride hexahydrate (CoCl_2_·6H_2_O, MW = 237.93 g/mol, 99.1% purity) were purchased from Merck. All solutions were prepared with Milli-Q water (deionized 0.22 µS, 25 °C, Millipore, Burlington, MA, USA).

### One-pot transformation of bCC to metal-doped apatites

Three 100 mL suspensions, each containing 490 mM of biogenic CaCO_3_ and 10 mM of either Co^2+^, Mg^2+^or Mn^2+^, and three 100 mL 300 mM KH_2_PO_4_ solutions were mixed (1:1 v/v) to obtain suspensions with stoichiometric (Ca + M)/P ratios. The process was repeated with an increased metal ion concentration to 20 mM. The experiments were performed over 7 days in an oven with circulating forced air using 10 mL Pyrex test tubes equipped with PBT screw caps and rubber discs coated with PTFE for temperatures ranging from 25 to 100 °C (Δ20 °C). For the range 120 °C ≤ T ≤ 200 °C (Δ20 °C), we used an aluminum box provided with a rack of PTFE tubes and a PTFE-coated aluminum cap to close the ensemble. In both temperature ranges, the percentage fill of the tubes was 70%. At the end of the experiments, the precipitates were washed four times by centrifugation (9000 rpm, 20 min each) with deionized water and then freeze-dried overnight.

### Particle characterization

XRD analysis was carried out using a Bruker D8 Advance Vario Series II powder diffractometer (Bruker AXS, Bruker GmbH, Karlsruhe, Germany) equipped with CuKα_1_ radiation (1.5406 Å) generator. Quantitative phase analysis was performed using the Rietveld method in the TOPAS 7 software (Bruker AXS, Bruker GmbH, Karlsruhe, Germany). FTIR spectra were recorded in transmittance mode with a Hyperion 3000 (Bruker, Massachusetts, USA) instrument in the wavenumber range from 4000 to 400 cm^−1^. The instrument was equipped with an attenuated total reflectance (ATR) accessory of diamond crystal. Raman spectra were recorded with a LabRAMHR spectrometer (Jobin–Yvon, Horiba, Tokyo, Japan) equipped with a laser diode emitting at a wavelength of 532 nm. CSD was analyzed by dynamic light scattering (DLS) with a Malvern Zetasizer Nano ZS analyzer (Malvern Instruments Ltd., Malvern, UK) using aqueous suspensions (~ 0.5 mg/mL, room temperature) contained in disposable polystyrene vials. For ζ-potential versus pH measurements, the pH of the suspensions was adjusted with HCl and NaOH solutions (0.25 and 0.1 M, respectively) as titration agents, without adding any additional electrolyte. For bCC particles the CSD measurements were performed by laser diffraction with a Mastersizer 2000 (Malvern, UK) particle size analyzer coupled to a Hydro 2000SM fully automated large volume wet sample dispersion unit.

TEM imaging was performed with a Libra 120 Plus TEM instrument (EELS) at 80 kV (Carl Zeiss, Jena, Germany). The samples were dispersed in absolute ethanol (99.8% v/v) and deposited on copper microgrids coated with a FORMVAR carbon film prior to observation. High-resolution TEM (HRTEM) analysis was performed with a TITAN G2 60-300 FEI Instrument (FEI, Hillsboro, OR, USA) operating at 300 kV. The instrument is equipped with EDX Super X detector to perform microanalysis and STEM type HAADF. For field emission scanning electron microscopy (FESEM) observations, we used a GEMINI LEO 1500 (Zeiss, Jena, Germany) instrument provided with an energy dispersive X-ray spectroscopy (EDX) analyzer by Oxford Instruments.

Elemental analysis of Mn and Co was carried out by inductively coupled plasma mass spectroscopy (ICP-MS) using a Perkin Elmer NexION 300D ICP Mass spectrometer (Perkin Elmer, Shelton, CT, USA). Ca, P and Mg were analyzed with Perkin Elmer ICP-OES OPTIMA 8300 spectrometer (Perkin Elmer, Shelton, CT, USA).

### Biological tests

#### Cells and cell cultures

MS1 cells, a murine pancreatic endothelial cell line, were purchased from ATCC (CRL-2279™) and were grown in Dulbecco’s modified Eagle’s medium (DMEM) (Sigma-Aldrich, St. Louis, MO, USA) supplemented with 10% fetal bovine serum (FBS), an antibiotic solution (streptomycin 100 µg/mL and penicillin 100 U/mL, Sigma-Aldrich, St. Louis, MO, USA) and 2 mM l-glutamine (complete medium). m17.ASC cells, an immortalized mouse mesenchymal stem cell clone from subcutaneous adipose tissue^44^, were grown and maintained in Claycomb medium (Sigma-Aldrich, St. Louis, MO, USA) supplemented with 10% fetal bovine serum (FBS), an antibiotic solution (streptomycin 100 µg/mL and penicillin 100 U/mL, Sigma-Aldrich, St. Louis, MO, USA) and 2 mM l-glutamine (complete medium). Cells were incubated at standard conditions (37 °C, 5% CO_2_) and split when at 80–90% confluency at a 1:10 ratio. Media were changed every two days. Murine osteoblasts progenitors (mOBPs) were isolated from C57BL/6 mouse (The Jackson Laboratory, no. 000664) as previously described^63^. Briefly, 3-week-old male mice (n = 9) were sacrificed by isoflurane (4%) inhalation followed by cervical dislocation, and their hind limbs were collected. Bone marrow cells were flushed out, and the cleaned bones (femurs, tibia, and humerus) were cut into small pieces and digested in a 1 mg/mL collagenase II (Sigma-Aldrich, St. Louis, MO, USA) solution at 37 °C for 2 h. The bone pieces were then plated in a flask with a basal medium containing αMEM (Gibco™, Thermo Fisher Scientific, Waltham, MA, USA), supplemented as previously described. The mOBPs emerging from the bone pieces were allowed to grow until they covered the plastic surface and reached confluence. Subsequently, after 3–5 in vitro passages, the cells were cultured for experimental procedures. C57BL/6 mice were maintained in a pathogen-free environment under regular settings. The Italian Health Ministry Authorization #758/2021-PR, project DB064.72, and the Animal Care and Use Committee of UPO, as well as the European Community Directive for Care and the Italian Laws on Animal Experimentation (Law by Decree 116/92), were followed in the approval of all operations. All methods were performed in accordance with the relevant guidelines and regulations. This study was conducted in accordance with the ARRIVE guidelines.

#### Cytotoxicity tests

To evaluate the cytotoxicity of the nanocomplexes, 6000, 5000 and 15,000 cells of MS-1, m17.ASC and mOBPs respectively, were seeded in 96-well plates and incubated for 24 h in their respective growth media. Different concentrations ranging from 0.1 to 100 µg/mL of sample were added in 100 µL of fresh medium. After 72 h of incubation, cell viability was evaluated by the (3-(4,5-dimethylthiazol-2-yl)-2,5-diphenyl tetrazolium bromide) (MTT) (Sigma-Aldrich, St. Louis, MO, USA) colorimetric assay. 20 µL of MTT solution (5 mg/mL in buffer PBS) was added to each well. After 2 h of incubation at 37 °C, the supernatants were removed and 125 µL of 0.2 N HCl in 2-propanol was added to dissolve the formazan crystals. 100 µL of each well were then transferred into a clean 96 well plate and the optical density was measured in a multiwell reader (2030 Multilabel Reader Victor TM X4, Perkin Elmer, Shelton, CT, USA) at 570 nm. The viability of untreated cells and their corresponding absorbance value were taken as 100% of cell viability, while values obtained from cells exposed to the different treatments were referred to this value. At least three experiments were performed using three replicates for each sample.

#### Cells and nanoparticles interactions

##### Cellular internalization by transmission electron microscope (TEM) and energy dispersive X-ray spectroscopy (EDX) analysis

m17.ASC and mOBPs cells (about 1 × 10^6^) were cultured as described above and incubated for 24 h with 25 µg/mL of particles. Following the procedures described by Oltolina et al.^64^, cells were fixed with 2.5% glutaraldehyde (Sigma Aldrich, St. Louis, MO, USA) and 2% paraformaldehyde (Sigma Aldrich, St. Louis, MO, USA) in PBS for one hour at 4 °C after being rinsed three times with PBS. Following three more rounds of washing with 0.1 M sodium cacodylate buffer, the samples were embedded in EMbed resin (Sigma Aldrich, St. Louis, MO, USA). Then, ultrathin slices (50–70 nm) were cut with a Reichert Ultracut S microtome (Leica Microsystems GmbH, Wetzlar, Germany), placed on copper grids, and stained with uranyl acetate and lead citrate following the protocol optimized by the laboratory technicians at the Scientific Instrumentation Centre (CIC) of the University of Granada. Images were acquired using a Talos F200C (Thermo Fisher Scientific, Waltham, MA, USA) Transmission Electron Microscope and acquired with Velox 3.12.1 (Thermo Fisher Scientific, Waltham, MA, USA). For EDX analysis, XFlash 6T/30 (Bruker Nano GmBH, Berlin, Germany) was used as detector and data were examined with Esprit 2.2 software (Bruker Nano GmBH, Berlin, Germany).

##### Cellular internalization by flow cytometry

m17.ASC and mOBPs cells (5 × 10^4^) were seeded in 12-well plate and after 24 h 25 µg/mL of NPs were added. After 1 and 7 days, cells were washed with PBS, detached with TryPle and resuspended in FACS buffer (2% FBS, 2 mM EDTA in PBS). For each sample, 1 × 10^5^ events were acquired, and the size and the granularity of cells were evaluated by Attune NxT Acoustic Focusing Cytometer (Thermo Fisher Scientific, Waltham, MA, USA) as described by Borroni et al.^65^. Data were examined by FlowJo™ v10 Software (Becton Dickinson, Life Science, Franklin Lakes, NJ, USA).

#### Osteogenic differentiation assays

##### Differentiation protocol

M17.ASC and mOBPs cells were seeded onto 6-well plates at a density of 1.5 × 10^5^ cells per well. After 24 h, cells were treated with an osteogenic medium (DMEM or αMEM containing 10% FBS, 50 μg/mL l-ascorbic acid-2-phosphate, 10 mM β-Glycerophosphate, and 10 nM dexamethasone) for 10–15 days. The osteoinductive effects of the nanoparticles were evaluated by co-culturing the cells along with 25 μg/mL of the sample in complete growth medium. The medium was carefully replaced every 3 days and samples were only added at the initial moment, to avoid accumulation issues.

##### Quantitative real-time PCR

The assessment of mRNA levels of genes was conducted through quantitative real-time PCR (q-RT-PCR) analysis. Initially, total RNA was extracted using Trizol (Invitrogen Life Technologies, Carlsbad, CA, USA). The concentration and quality of the RNA were subsequently evaluated using the NanoDrop 2000C spectrophotometer (Thermo Fisher Scientific, Waltham, MA, USA). Following RNA purification and DNAse I treatment (Thermo Fisher Scientific, Waltham, MA, USA), 1 µg of RNA was reverse transcribed into complementary DNA (cDNA) using the RevertAidTM H Minus First Strand cDNA Synthesis Kit (Thermo Fisher Scientific, Waltham, MA, USA) with oligo(dT) primers. Gene assays were performed in triplicate for each treatment within a 12 µL reaction volume, consisting of 1 µL of the cDNA products, 6 µL of Sso-Fast EVA Green SMX (Bio-Rad, Hercules, CA, USA), and 500 nM of both forward and reverse primers, as detailed in Table S3.

The q-RT-PCR was carried out using an automated CFX96 real-time thermocycler (Bio-Rad, Hercules, CA, USA) with the following cycling conditions: an initial denaturation at 95 °C for 1 min, followed by 45 cycles at 98 °C for 5 s, and an annealing/extension step for 5 s at 60 °C, with data collection. Subsequently, a melting curve analysis was performed at the end of these cycles, ranging from 65 to 95 °C, with a plate read every 0.5 °C. This step was crucial for verifying the specificity of the amplification product by confirming a single-peak melting temperature. The obtained results were analysed using the Bio-Rad CFX Manager software, and the gene expressions were calculated employing the DDCt method, with β-actin serving as the internal control for normalization.

##### Alkaline phosphatase staining and quantitative analysis

To evaluate the osteogenic differentiation at 14 days of treatment, alkaline phosphatase (ALP) staining was carried out as described in Dupont et al.^66^. m17.ASC cells and mOBPs were washed three times with PBS, fixed with 4% paraformaldehyde (4% PFA; Sigma-Aldrich, St. Louis, MO, USA) for 15 min and stained with an alkaline phosphatase detection kit (Millipore, Merk Millipore, Mi, Italy). Optical microscopy at 200× magnification was used to analyze and photograph the samples. ALP quantification was performed by measuring the violet intensity of the staining using ImageJ software. Experiments were performed at least three times.

##### Alizarin red staining (ARS) and quantitative analysis

Alizarin Red staining was used to evaluate the calcium phosphate deposition of m17.ASC cells and mOBPs after 14 days of treatment with the nanoparticles. Cells were washed with PBS pH 7.2, fixed with PFA (2 wt% in PBS) and stained with Alizarin Red Solution (40 mM, pH 4.1) for 30 min at room temperature. To remove non-specific precipitate, stained cells were washed thrice with bi-distilled water. Then, optical microscopy at 200× magnification was used to analyze and photograph the samples. Calcium phosphate deposits were quantified by dissolving the staining with 10% acetic acid (Sigma-Aldrich, St. Louis, MO, USA) for 30 min. 150 μL of each sample were then transferred into a 96-well plate and the optical density was measured in a multiwell reader (2030 Multilabel Reader Victor TM X4, Perkin Elmer, Shelton, CT, USA) at 405 nm. Experiments were performed at least three times.

##### Statistical analysis

GraphPad Prism version 10.2.3 for Windows, GraphPad Software (GraphPad Prism, San Diego, CA, USA) was used to perform the statistical analysis. The results were expressed as mean ± standard deviation of three triplicates, using a one-way ANOVA with the appropriate post-test for grouped analyses. Differences at *p* < 0.05 (*), *p* < 0.01 (**), *p* < 0.001 (***), and *p* < 0.0001 (****) were statistically significant.

## Electronic Supplementary Material

Below is the link to the electronic supplementary material.

Supplementary Material 1

## Data Availability

The authors declare that the data supporting this study’s findings are available within the paper and its Supplementary Information files. Should any raw data files be needed in another format, they are available from the corresponding author upon reasonable request.
