# Supplementary material for: Sustainable production of osteoinductive Co2+, Mg2+ and Mn2+ -substituted apatites particles by one-pot conversion of biogenic calcium carbonate
Source: Sci Rep. 2025 Mar 29;15:10893. doi: 10.1038/s41598-025-94792-7 (PMC11954947; doi:10.1038/s41598-025-94792-7)
Supplement: Supplementary file 1 — Supplementary Material 1 [file 41598_2025_94792_MOESM1_ESM.pdf]

Supplementary Information

Sustainable preparation of osteoinductive Co^{2+}, Mg^{2+} and Mn^{2+} -substituted apatites particles by one-pot conversion of biogenic calcium carbonate

Sandra María Cano-Plá[a], Francesca Oltolina[b], Francisco Javier Acebedo-Martínez[a] Raquel Fernández-Penas[a], Cristóbal Verdugo-Escamilla[a], Carla Triunfo[c,d], Paolo Emanuele Di Simone[b], Chiara Borsotti[b], Antonia Follenzi[b]*, Gabriele Maoloni[e], Giuseppe Falini[c], Jaime Gómez-Morales[a]*

[a]*Laboratory of Crystallographic Studies, Andalusian Earth Science Institute, Spanish National Research Council, Avda. Las Palmeras, nº 4, 18100 Armilla (Spain)*

[b]*Dipartimento di Scienze della Salute, Università del Piemonte Orientale, "A. Avogadro" Via Solaroli 17, 28100 Novara (Italy).*

[c]*Department of Chemistry "Giacomo* Ciamician*", University of Bologna, via F. Selmi 2, 40126 Bologna (Italy).*

[d]*Fano Marine Center. The Inter-Institute Center for Research on Marine Biodiversity, Resources and Biotechnologies, Viale Adriatico 1/N 61032 Fano (Italy).*

[e]*Plant Ascoli Piceno, Finproject S.p.A.,3100 Ascoli Piceno, Italy*

*Corresponding authors: jaime.gomez@csic.es (*J. G.-M.*), antonia.follenzi@med.uniupo.it (*A.F.*)

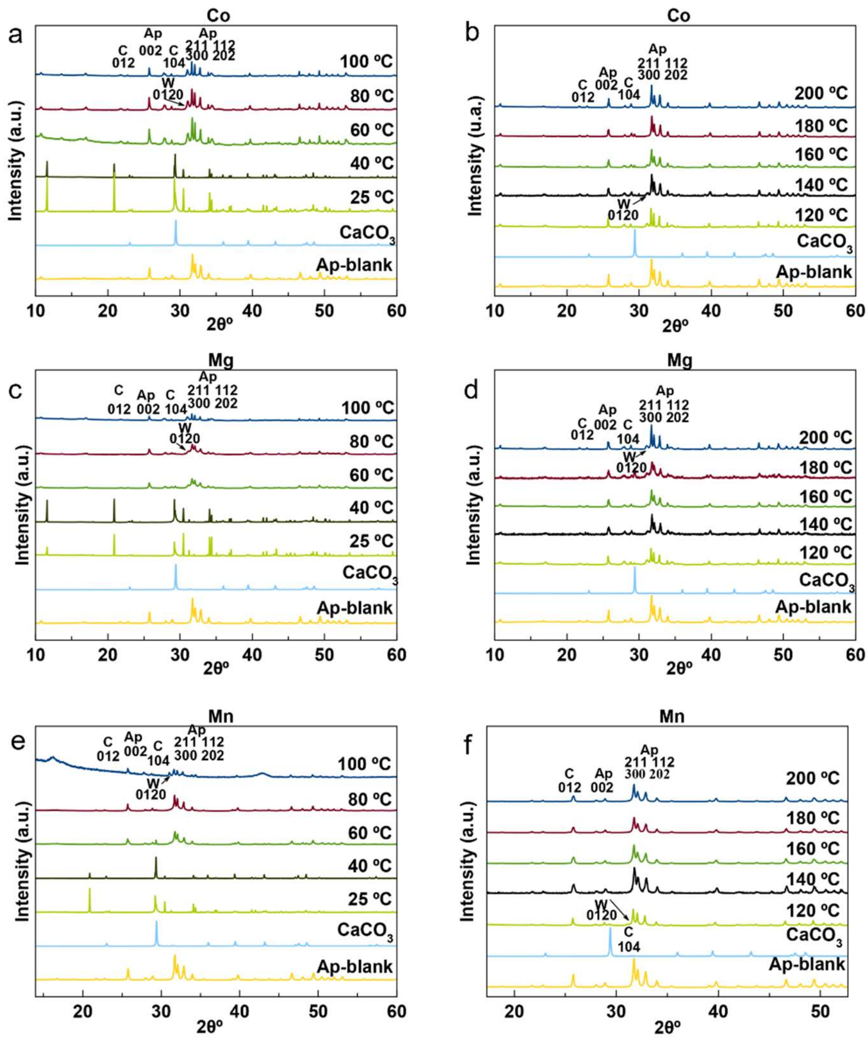

Figure S1. XRD patterns of Ap samples prepared from biogenic $CaCO_3$ at different temperatures (from 25 °C to 200 °C) in presence of 10 mM of Co^{2+} (a, b), Mg^{2+} (c, d) and Mn^{2+} (e, f).

Table S1. Chemical composition (ppm), (Ca+M)/P molar ratios determined by ICP-MS and ICP-OES, and phase composition determined by Rietveld refinement of diffractograms of samples prepared at 200 °C using 10 mM of M dopant

**M, (dopant)**	Co (ppm)	Mg (ppm)	Mn (ppm	Ca (ppm)	P (ppm)	(Ca+M/P (mol)	Ap-M (wt%)	MW (wt%)	C (wt%)
**-**	0	0	0	297.2	127,10	1.82	99.4	0	0.6
**Co^{2+}**	7.7	0	0	287.2	135.70	1.66	94.7	5.3	0
**Mg^{2+}**	0	4.3	0	286.7	136.30	1.66	70.1	28.7	1.2
**Mn^{2+}**	0	0	1.1	280.3	127.2	1.71	98.4	0	1.6

Ap-M (metal-doped apatite); M-W (metal-doped whitlockite); C (calcite)

Table S2. Phase composition (wt%) of samples obtained at 120 ºC, 160 ºC and 200 ºC in presence of 10 mM dopant metal

**Metal**	**Phase**	**120 ºC**	**160 ºC**	**200 ºC**
**Co**	M-Ap	56.7	83.2	94.7
	M-W	38.8	13.1	5.3
	bCCP	4.5	3.7	0
**Mg**	M-Ap	57.6	85.8	70.1
	M-W	39.1	12.3	28.7
	bCCP	3.3	1.9	1.2
**Mn**	M-Ap	86.7	97.9	98.4
	M-W	10.0	0	0
	bCCP	4.3	2.1	1.6

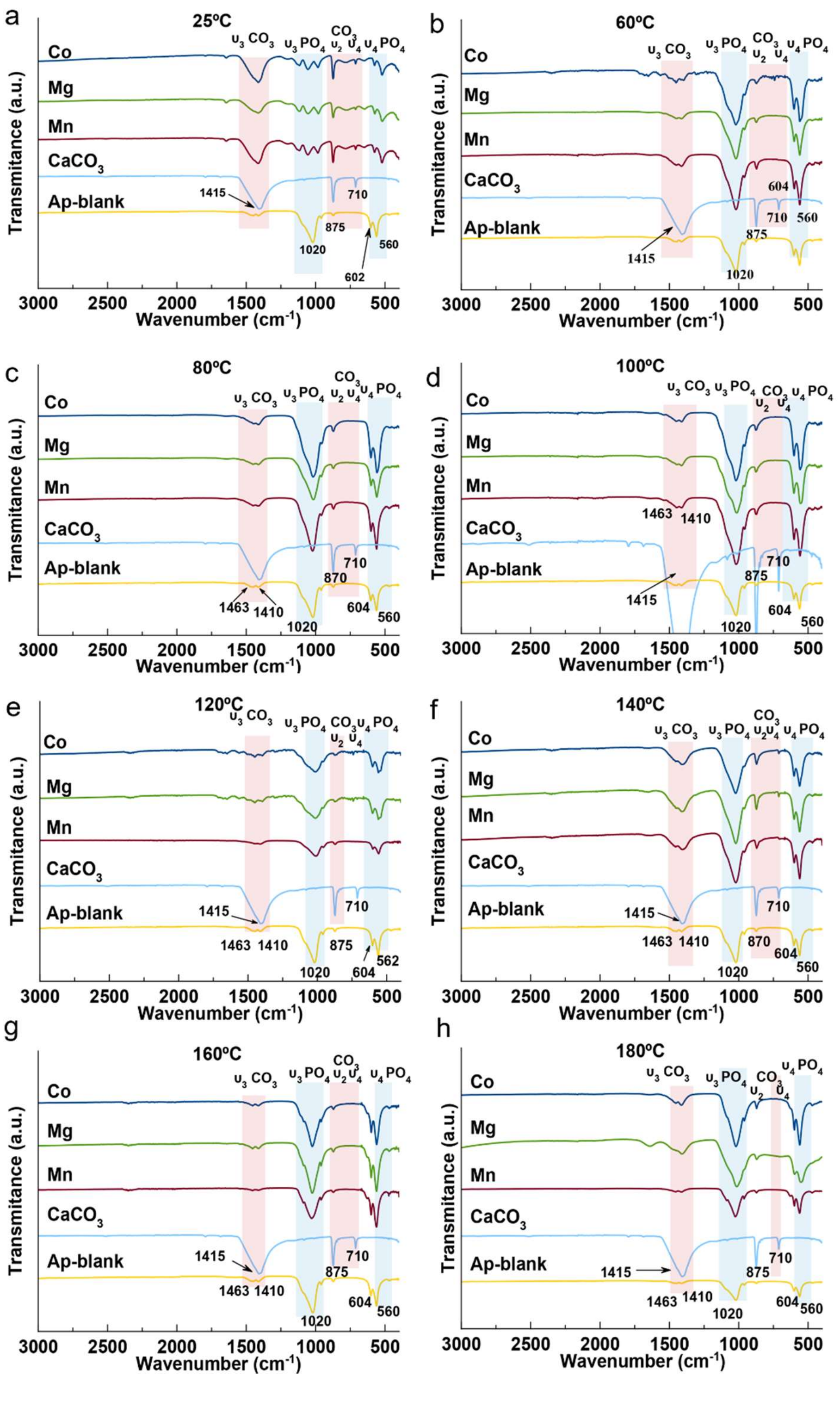

Figure S2. FTIR spectra of metal-doped CaP samples prepared from biogenic $CaCO_3$ in presence of 10 mM of Mn^{2+}, Mg^{2+} and Co^{2+} at 25 °C (a), 60 °C (b), 80 °C (c), 100 °C (d), 120 °C (e), 140 °C (f), 160 °C (g) and 180 °C (h).

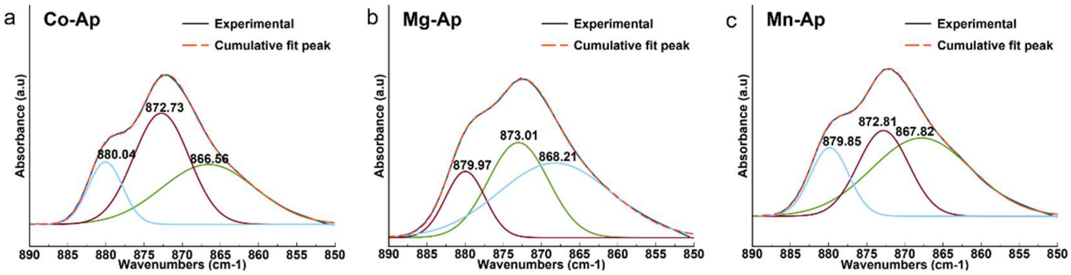

Figure S3. Deconvolution of the FTIR $\nu_2CO_3^{2-}$ band centered at ~ 875 cm^{-1} of Ap-Co (a), Ap-Mg (b), and Ap-Mn (c) samples prepared from bCCP in the presence of 10 mM dopant metal at 200 °C.

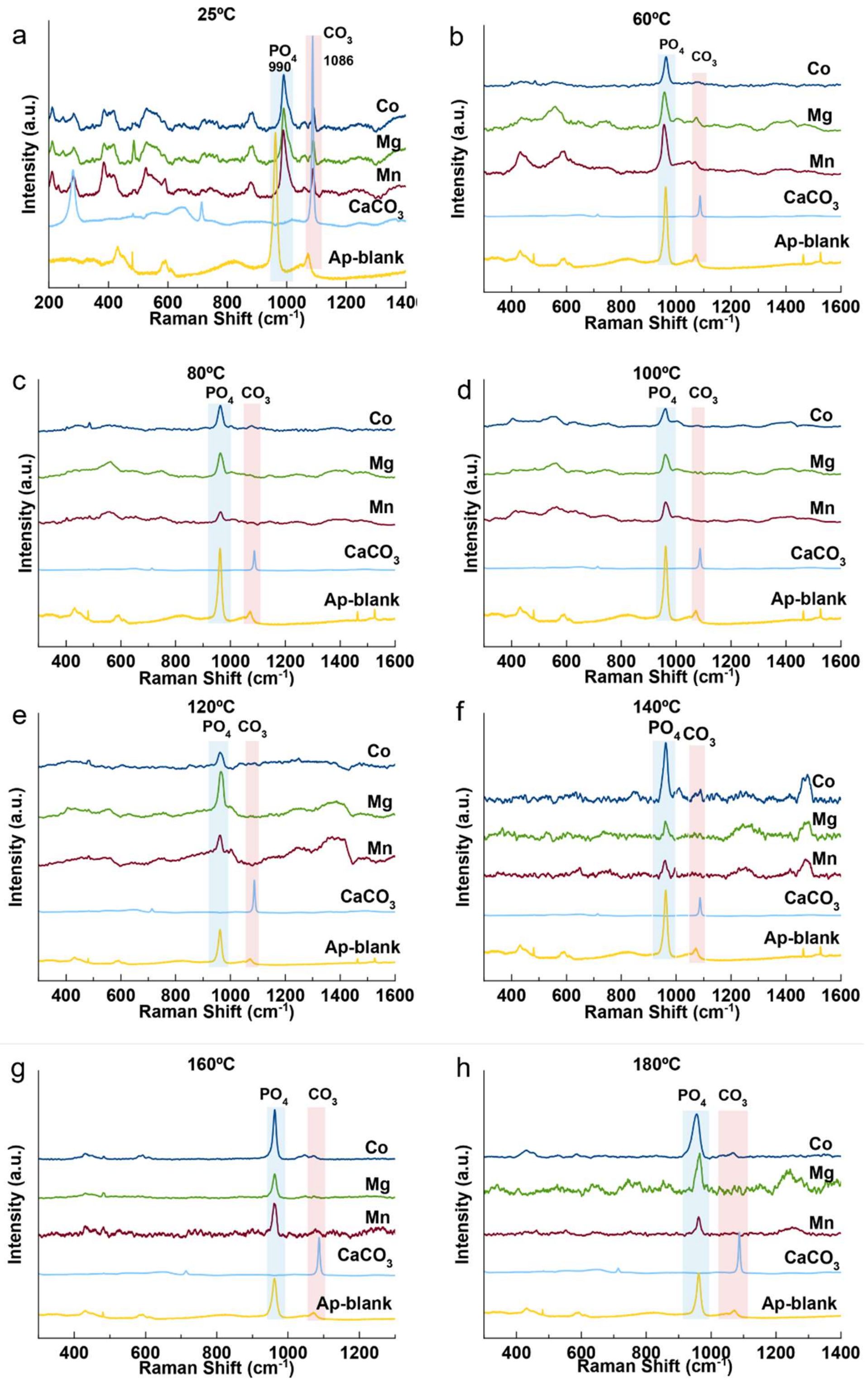

Figure S4. Raman spectra of metal-doped Ap samples prepared from biogenic $CaCO_3$ in the presence of 10 mM of Mn^{2+}, Mg^{2+} or Co^{2+} at 25 °C (a), 60 °C (b), 80 °C (c), 100 °C (d), 120 °C (e), 140 °C (f), 160 °C (g) and 180 °C (h).

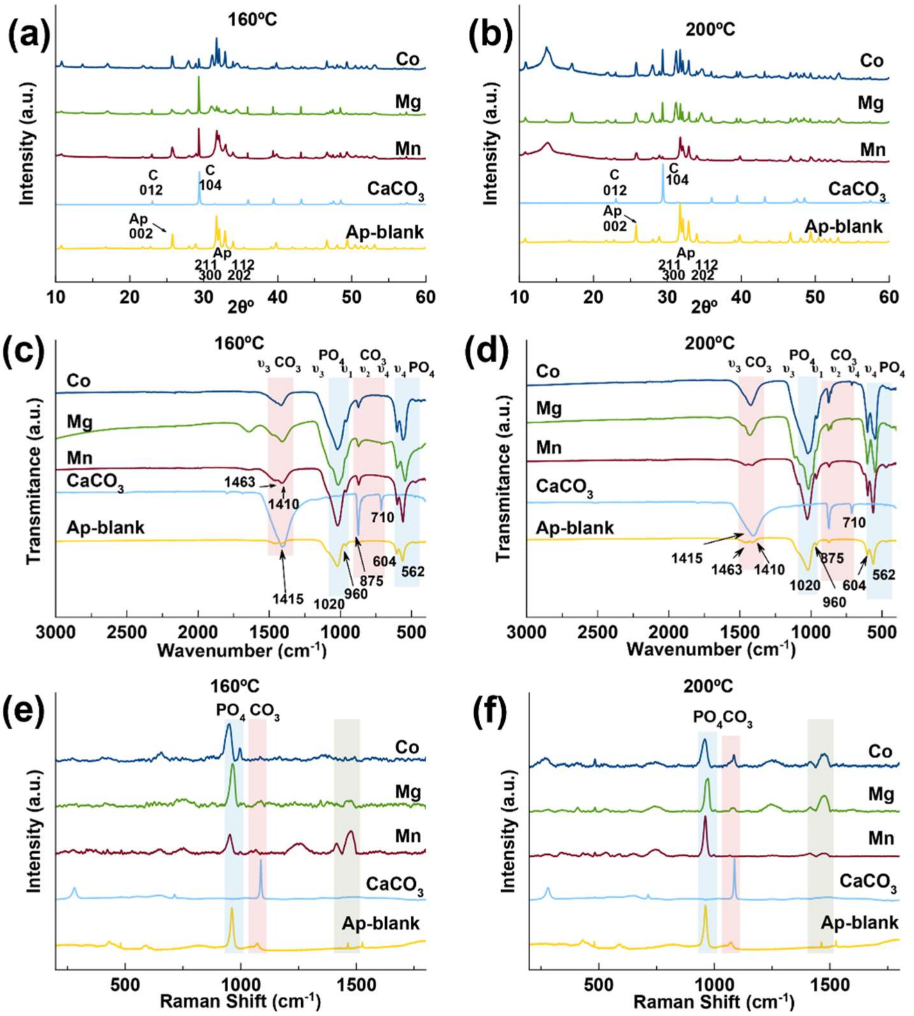

Figure S5. XRD patterns (a, b), FTIR (c, d), and Raman spectra (e, f) of metal-doped Ap samples prepared from biogenic $CaCO_3$ at 160 °C and 200 °C in the presence of 20 mM of Mn^{2+}, Mg^{2+} or Co^{2+} ions.

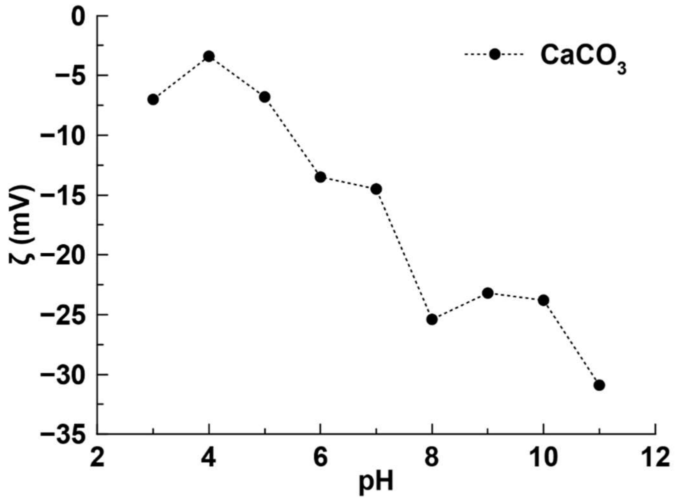

Figure S6. ζ-potential versus pH of bCCP

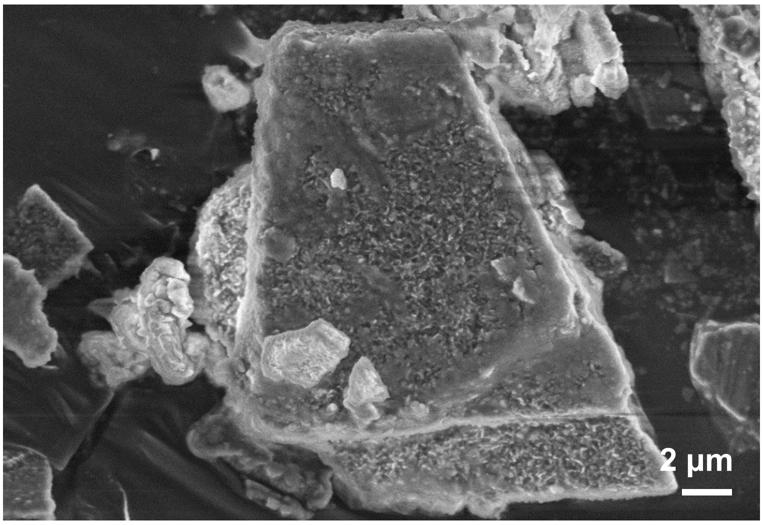

Figure S7. FESEM image of biogenic $CaCO_3$ particles milled and sieved at Ø <45 µm

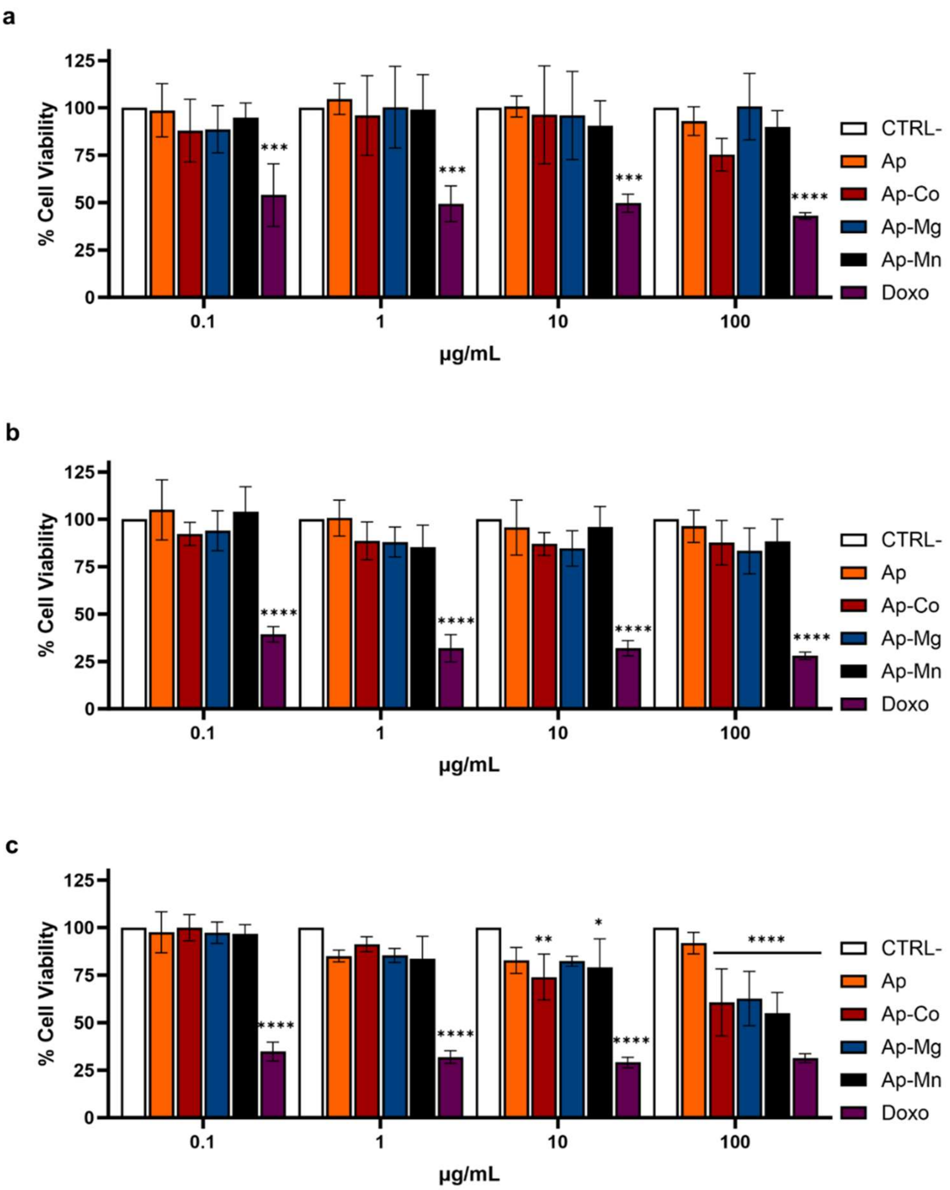

Figure S8. Cell viability of Ap, Ap-Co, Ap-Mg, Ap-Mn particles, as well as Doxo, evaluated in MS1 (a), m17.ASC (b) and mOBPs (c) cells, compared to untreated samples. Statistical significance was determined using Dunnett's multiple comparisons test (* $p < 0.05$; ** $p < 0.01$; *** $p < 0.001$; **** $p < 0.0001$)

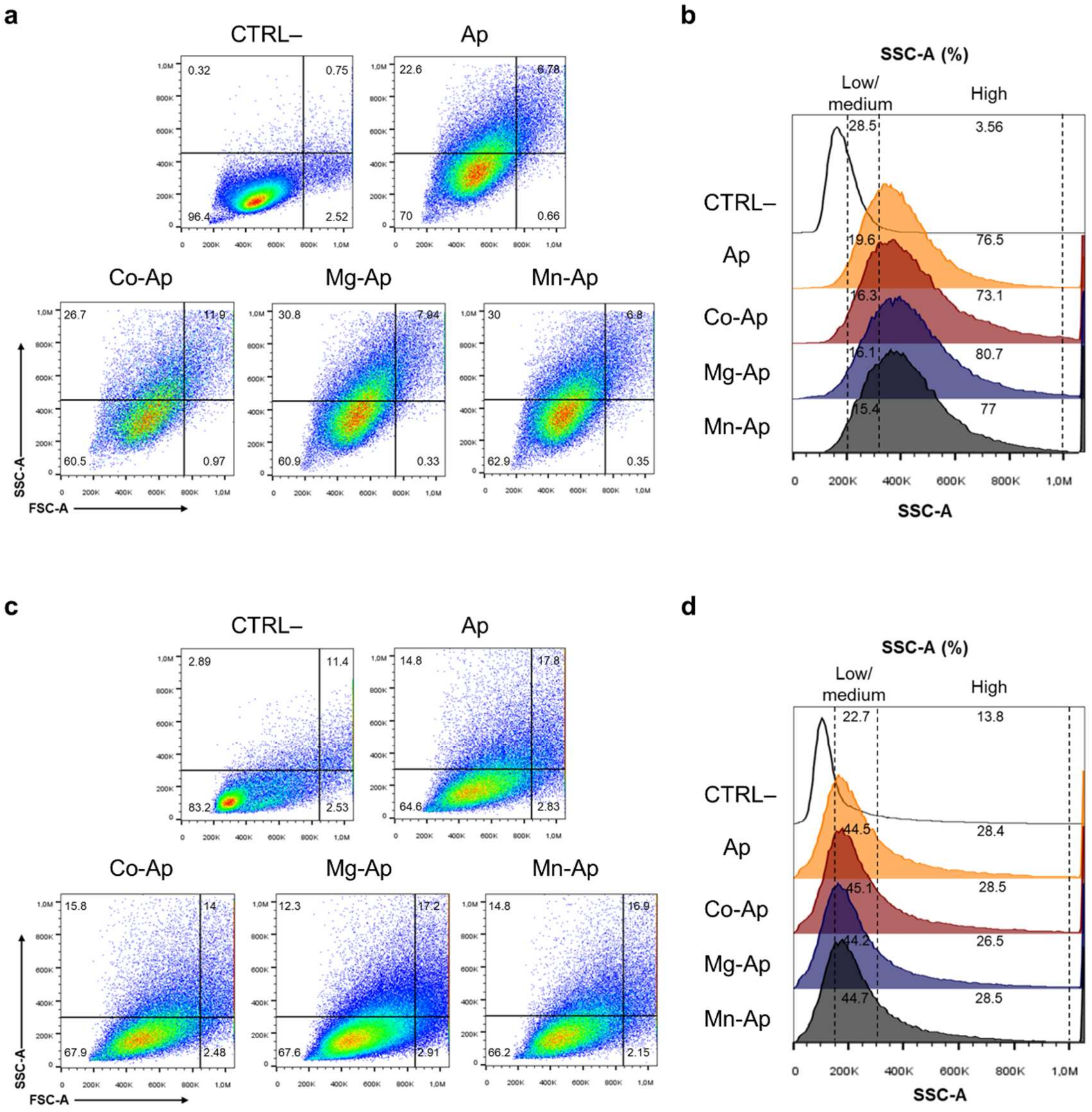

Figure S9. Interaction of metal-doped Ap samples with m17.ASC and mOBPs cells after 7 days analyzed by flow cytometry. Representative dot plots show changes in morphology, based on physical parameters such as size (FSC-A) and granularity/complexity (SSC-A), in m17.ASC (a) and mOBPs (c) cells treated with the particles, compared to untreated controls (CTRL-). Representative histograms display the percentage of cells with increased granularity/complexity, categorized by low/medium, or high SSC-A, for (b) m17.ASC and (d) mOBPs cells relative to the untreated control (CTRL-).

Table S3. Sequences of the primers used for q-RT-PCR.

Target gene	Forward Sequence	Reverse Sequence
**BMP2**	GGGACCCGCTGTCTTCTAGT	TCAACTCAAATTCGCTGAGGAC
**COL1A1**	CCCCAACCCTGGAAACAGAC	GGTCACGTTCAGTTGGTCAAAGG
**COL1A2**	CCTCCGTCTACTGTCCACTGA	ATTGGAGCCCTGGATGAGCA
**BGLAP**	GGCCCTGAGTCTGACAAAGC	GCTCGTCACAAGCAGGGTTAA
**SPP1**	AGCAAGAAACTCTTCCAAGCAA	GTGAGATTCGTCAGATTCATCCG
**RANKL**	TGTACTTTCGAGCGCAGATG	AGGCTTGTTTCATCCTCCTG
**β-actin**	GATGACCCAGATCATGTTTGA	GGAGAGCATAGCCCTCGTAG

Target genes of bone morphogenetic protein 2 (BMP2), type 1 collagen A (COL1A1 – COL1A2), bone gamma-carboxyglutamate protein (BGLAP – osteocalcin), secreted phosphoprotein 1 (SPP1 - osteopontin) and receptor activator of nuclear factor kappa B ligand (RANKL).
